# Intelligent connected adaptive signal control considering pedestrians based on the EXP-DDQN algorithm

**DOI:** 10.1371/journal.pone.0322945

**Published:** 2025-06-06

**Authors:** Sen Cao, Yaping Sun, Xingchen Zhang, Mengyang Yang

**Affiliations:** 1 School of Traffic Engineering, Huanghe Jiaotong University, Jiaozuo, China; 2 School of Traffic and Transportation, Beijing Jiaotong University, Beijing, China; University of Shanghai for Science and Technology, CHINA

## Abstract

With the increasing integration of Connected and Automated Vehicles (CAVs) and Human-Driven Vehicles (HDVs) in urban traffic systems, along with highly variable pedestrian crossing demands, traffic management faces unprecedented challenges. This study introduces an improved adaptive signal control approach using an enhanced dual-layer deep Q-network (EXP-DDQN), specifically tailored for intelligent connected environments. The proposed model incorporates a comprehensive state representation that integrates CAV-HDV car-following dynamics and pedestrian flow variability. Additionally, it features an improved MC Greedy exploration strategy and prioritized experience replay, enabling efficient learning and adaptability in highly dynamic traffic scenarios. These advancements allow the system to dynamically adjust green light durations, phase switches, and pedestrian phase activations, achieving a fine balance between efficiency, safety, and signal stability. Experimental evaluations underscore the model’s distinct advantages, including a 26.9% reduction in vehicle-pedestrian conflicts, a 31.83% decrease in queue lengths, a 32.52% reduction in delays compared to fixed-time strategies, and a 35.17% reduction in pedestrian crossing wait times. Furthermore, EXP-DDQN demonstrates significant improvements over traditional DQN and DDQN methods across these metrics. These results underscore the method’s distinct capability to address the complexities of mixed traffic scenarios, offering valuable insights for future urban traffic management systems.

## Introduction

With the continuous growth of urban traffic demand and the increasing diversity of vehicle types, traditional signal control methods are becoming increasingly inadequate in addressing the challenges posed by mixed traffic flows (including both autonomous and human-driven vehicles) and pedestrian crossing demands. On the one hand, Connected and Automated Vehicles (CAVs) can acquire detailed traffic information through Vehicle-to-Vehicle (V2V) and Vehicle-to-Infrastructure (V2I) communication, enabling more precise acceleration, deceleration control, and driving strategies. On the other hand, Human-Driven Vehicles (HDVs) exhibit significant differences in driver reaction times, randomness, and car-following behavior. The mixed flow of these two types of vehicles significantly increases the complexity of intersection traffic operations [1,2]. Meanwhile, in urban central areas, pedestrian flow peaks vary significantly between weekdays and weekends. If pedestrian crossing demands are not promptly met, issues such as prolonged waiting times or random jaywalking may arise, severely impacting traffic safety and efficiency [3]. Therefore, dynamically optimizing signal control by leveraging intelligent connected technologies while fully considering the characteristics of pedestrian crossing and mixed traffic flows has become a critical challenge in urban traffic management.

Traffic signal control optimization is an essential means of improving the operational efficiency of urban transportation infrastructure, particularly in alleviating congestion and enhancing traffic flow [4]. Based on different control strategies, existing traffic signal control methods can be categorized into three types: fixed-time control, actuated control, and adaptive control [5]. Fixed-time control divides signal phases into fixed time intervals and is suitable for roads with relatively stable traffic flows. Actuated control dynamically adjusts signal timings based on real-time traffic demands, making it ideal for environments with rapidly changing traffic conditions. Adaptive control, on the other hand, dynamically adjusts signal strategies by acquiring real-time traffic flow data and applying optimization algorithms, and is widely used in complex traffic networks, such as SCOOT and SCATS [6].

The rapid development of artificial intelligence, especially reinforcement learning (RL) methods, has introduced new opportunities to the field of traffic signal control. Reinforcement learning is an intelligent algorithm that interacts with the environment to optimize strategies through learning experiences rather than relying on fixed-parameter models [7]. In recent years, the successful application of deep learning and reinforcement learning in traffic signal control has inspired extensive research into adaptive control for intelligent connected mixed traffic flows. Some studies have focused on multi-agent architectures, achieving real-time optimization of coordinated signal timings across networks in simulation environments. Others have concentrated on complex scenarios at single intersections, incorporating the demands of various vehicle types and pedestrians into state and reward designs, significantly reducing queues and delays during peak periods [8,9].

Existing literature indicates that increasing the proportion of autonomous vehicles (CAV penetration rate) can not only improve traffic efficiency but also reduce potential conflicts through vehicle-road coordination. However, under mixed traffic conditions, signal control strategies must balance multiple factors, including HDV start-up delays, CAV collaborative acceleration, and pedestrian-specific phases, to improve overall travel efficiency without compromising safety [10,11]. Pedestrian crossing demands, in particular, cannot be overlooked: if signal timing neglects pedestrian flow characteristics, excessive waiting times may lead to safety risks and induce potential red-light violations, further deteriorating intersection performance [12].

Extensive research has been conducted on optimizing pedestrian crossing signals and traffic flow. Some studies focus on pedestrian-vehicle conflict theories and statistical modeling, using metrics such as Time-to-Collision (TTC) or Post Encroachment Time (PET) to assess potential pedestrian-vehicle conflict risks [13,14]. Other research emphasizes pedestrian waiting times and flow characteristics, aiming to balance pedestrian comfort and vehicle throughput by setting minimum green times, pedestrian-priority phases, or multi-modal signal phases [15]. However, under traditional timing or rule-based algorithm frameworks, signal control often fails to dynamically adapt to rapidly changing mixed traffic flows and unstable pedestrian flow characteristics. When the penetration rate of autonomous vehicles gradually increases while a significant proportion of traditional vehicles and pedestrians still coexist, fixed or semi-fixed signal timing becomes even less capable of meeting actual traffic demands [16].

In recent years, leveraging deep reinforcement learning algorithms and multi-agent architectures, some scholars have incorporated multi-modal information, such as pedestrian states (e.g., the number of pedestrians queued and their waiting times), as well as vehicle queue lengths, speeds, and proportions, into signal control systems. Experiments at single or multiple intersections have demonstrated certain successes. The basic approach involves embedding the conflict relationships between pedestrian crossing behavior and vehicle flow into the state vectors and reward functions, enabling agents to actively balance pedestrian and vehicle efficiency while prioritizing safety during the reinforcement learning process [17].

While adaptive control strategies can better accommodate fluctuating traffic demands, most existing methods still rely on model-based algorithms. These methods often assume idealized traffic models (e.g., uniform traffic arrival rates) and pre-set parameters, limiting their practical effectiveness. To overcome these challenges, researchers have been exploring new algorithmic frameworks to enhance the real-world performance of adaptive traffic signal control methods. By integrating deep learning with reinforcement learning, Deep Reinforcement Learning (DRL) algorithms have been proposed, leveraging deep neural networks (such as convolutional neural networks [CNN] and recurrent neural networks [RNN]) to enhance model representation capabilities [18], thereby addressing the shortcomings of traditional methods in complex environments.

This study proposes an enhanced exploration strategy-based dual-layer deep Q-network model (EXP-DDQN). Compared to traditional DQN algorithms, this approach combines the strengths of Deep Q-Networks (DQN) and Double DQN to address the overestimation issues inherent in traditional Q-learning methods. By introducing a decoupled update mechanism for target and evaluation networks, DDQN reduces Q-value overestimation bias, improving the stability and reliability of signal control decisions. Furthermore, our algorithm design incorporates adaptations to complex traffic environments, enabling a more precise evaluation of the effectiveness of traffic signal control.

## Materials and methods

### State function

**Fig 1:**
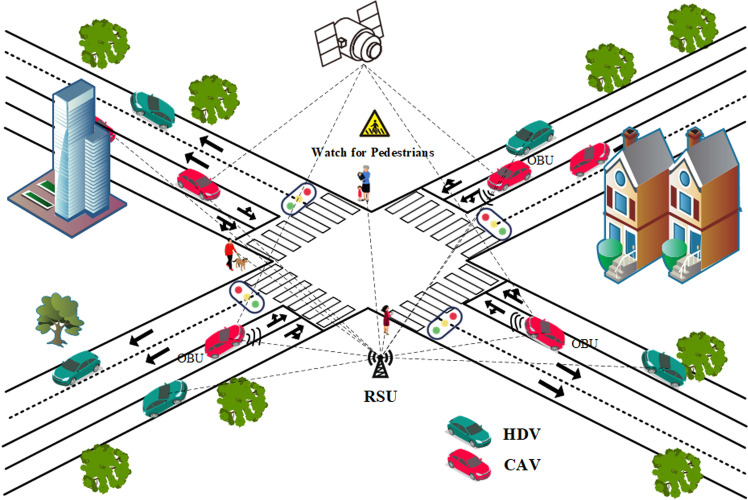
Intelligent networked adaptive signal control for pedestrian crossing.


In the context of an intelligent connected environment, the pedestrian crossing scenario is illustrated in Fig 1. The figure depicts a typical intelligent traffic interaction system, involving various participants such as vehicles, pedestrians, and roadside units (RSUs). RSUs are responsible for communicating with vehicle and pedestrian devices, managing traffic signals, enabling information exchange, and collecting data. On-board units (OBUs), installed in vehicles, receive signals and information transmitted by RSUs while simultaneously sending dynamic vehicle data back to the system center. To characterize the traffic operation at intersections in a mixed traffic environment while simultaneously addressing pedestrian crossing demands, this paper defines the system state at time *t* as a vector composed of three core sub-states:

$$S_{t}=\left(S_{t}^{\left(h\right)},S_{t}^{\left(c\right)},S_{t}^{\left(p\right)},S_{t}^{\left(l\right)}\right)$$ (1)

Where $S_{t}^{\left(h\right)}$ represents the state of Human-Driven Vehicles (HDVs); $S_{t}^{\left(c\right)}$ represents the state of Connected and Automated Vehicles (CAVs); $S_{t}^{\left(p\right)}$ represents the state of pedestrians; $S_{t}^{\left(l\right)}$ represents the state of traffic light.

Considering the queuing dissipation characteristics, speed distribution, and car-following behavior of HDVs, the HDV state at time *t*, $S_{t}^{\left(h\right)}$ is expressed as:


$$S_{t}^{\left(h\right)}=\left(Q_{t}^{\left(h\right)},V_{t}^{\left(h\right)},X_{t}^{\left(h\right)}\right)$$ (2)

Where $Q_{t}^{\left(h\right)}$ and $V_{t}^{\left(h\right)}$ represent macroscopic metrics describing the queuing size and speed distribution of HDVs, respectively; $X_{t}^{\left(h\right)}$ is a key microscopic parameter used to describe the car-following behavior of HDVs, capturing the dynamic characteristics of queue dissipation, acceleration, and deceleration responses.

In many microscopic traffic flow studies, the Intelligent Driver Model (IDM) is a commonly used car-following model for HDVs [19]. The IDM assumes that the acceleration of each vehicle, $\dot{v}(t)$ depends on its current speed v(t), the distance to the vehicle ahead s(t), and the speed difference  Δ v(t) between the two vehicles. The basic form of the IDM is as follows:


$$\dot{v}(t)=a\left[1-{\left(\frac{v(t)}{v_{0}}\right)}^{\delta }-{\left(\frac{s^{*}(v(t),\;\;\;\Delta \;\;\;v(t))}{s(t)}\right)}^{2}\right]$$ (3)

$$s^{*}(v,\;\;\;\Delta \;\;\;v)=s_{0}+vT+\frac{v\;\;\;\Delta \;\;\;v}{2\sqrt{ab}}$$ (4)

Where: $s_{0}$ is the minimum safe headway distance; T is the desired time headway; a and b are the maximum acceleration and comfortable deceleration respectively; δ is an exponent typically set to 4; and $v_{0}$ is the desired speed of the vehicle.

To emphasize the focus on queue dissipation and headway distance, this paper defines $X_{t}^{\left(h\right)}$ as follows:


$$X_{t}^{\left(h\right)}=\left[\begin{array}{c} s_{t,1}^{\left(h\right)},\;\;\;\Delta \;\;\;v_{t,1}^{\left(h\right)},{\hat{\tau }}_{t,1}^{\left(h\right)},\ldots ,s_{t,i}^{\left(h\right)},\;\;\;\Delta \;\;\;v_{t,i}^{\left(h\right)},{\hat{\tau }}_{t,i}^{\left(h\right)} \end{array}\right]$$ (5)

Where $s_{t,i}^{\left(h\right)}$ represents the average or typical headway distance for HDVs in lane *i*; $\;\;\;\Delta \;\;\;v_{t,i}^{\left(h\right)}$ reflects the speed difference between HDVs in lane *i* and their preceding vehicles, capturing the intensity of acceleration or deceleration behavior; ${\hat{\tau }}_{t,i}^{\left(h\right)}$ approximates queue start-up delays and driver reaction times.

ACC and CACC are commonly used car-following models in real-time cruise control systems for vehicles [20]. ACC is a cruise control system that adjusts a vehicle’s speed based on the speed of the preceding vehicle. Its car-following models typically use either a linear headway distance model or a second-order headway distance model. The linear model assumes that the vehicle spacing is proportional to the speed, while the second-order model assumes that the spacing is inversely proportional to the square of the speed.

CACC is an advanced version of ACC, characterized by vehicle-to-vehicle communication for coordination. The CACC car-following model typically employs the CACC-MODE-X model, which considers not only the spacing and speed between vehicles but also the acceleration and the preceding vehicle’s acceleration. Compared to ACC, CACC offers higher following accuracy and shorter reaction times. Additionally, through vehicle-to-vehicle communication, CACC can coordinate platoon driving, thereby enhancing safety, efficiency, and comfort within the platoon.

In autonomous vehicles, the trailing vehicle leverages dynamic information exchange technology to understand the driving state of the lead vehicle and surrounding road conditions. By dynamically capturing road information, vehicles achieve coordinated real-time driving on the road [21].

The state of Connected and Automated Vehicles (CAVs) at time *t* is defined as:


$$S_{t}^{\left(c\right)}=\left(Q_{t}^{\left(c\right)},V_{t}^{\left(c\right)},X_{t}^{\left(c\right)}\right)$$ (6)

Where $Q_{t}^{\left(c\right)}$ represents the queue length of CAVs in each lane; $V_{t}^{\left(c\right)}$ denotes the average speed of CAVs in each lane; $X_{t}^{\left(c\right)}$ captures the microscopic coordination characteristics of CAVs as governed by the CACC and ACC car-following models.

In the CACC car-following model, speed control and distance control are conducted separately. Speed control adjusts vehicle acceleration and speed based on the distance and speed difference between vehicles, ensuring smooth following of the leading vehicle at a consistent speed. Distance control manages the spacing between vehicles, maintaining a safe following distance. The PATH laboratory validated the CACC car-following model through small-scale platoon experiments, as described in Equations (7) and (8):


$$v_{s}=v_{s-1}+r_{p}\cdot e_{s-1}+r_{d}\cdot {\overset{⬝}{e}\;}_{s-1}$$ (7)

$$e_{s}=x_{\text{c}}-x_{m}-x_{\Delta }-\tau_{c}\cdot v_{s-1}$$ (8)

Where $v_{s}$ is the velocity of the target vehicle at time step *t*; $v_{s\text{-}1}$ is the velocity of the *t*arget vehicle at the previous time step; $e_{s}$ is the gap error of the target vehicle at time step *t*; $e_{s\text{-}1}$ is the gap error of the target vehicle at the previous time step; $r_{p}$ is a coefficient, typically 0.45 s^−1^; $r_{d}$ is a coefficient, *t*ypically 0.25; $x_{c}$ is the current inter-vehicle distance; $x_{m}$ is the minimum stopping distance; $x_{\Delta }$ is the length of the vehicle; $\tau_{c}$ is the desired time headway for the CACC car-following model.

For HDVs, the desired headway distance is $\tau_{V}$, while for CAVs, it is $\tau_{B}$. Assuming the inter-vehicle gap error is zero and the vehicle speed is constant as the CAV passes through an intersection, the minimum headway distance $x_{cB}$ to the preceding vehicle can be expressed as Equation (9):


$$x_{cB}=v_{s}\tau_{B}+x_{\Delta }+x_{m}$$ (9)

For HDVs passing through an intersection, assuming a zero inter-vehicle gap error and a start-up time of t_0_, the minimum headway distance $l_{cV}$ to the preceding vehicle can be expressed as Equation (10):


$$e_{s}=x_{\text{c}}-x_{m}-x_{\Delta }-\tau_{c}\cdot v_{s-1}$$ (10)

Where $a_{s}$ represents the acceleration of the CACC vehicle at the current time step.

The primary advantage of the CACC car-following model lies in its ability to achieve coordinated control within a vehicle platoon, significantly enhancing transportation efficiency and safety. Furthermore, the CACC model can reduce traffic congestion and carbon emissions, contributing to safer and more sustainable roadways.

The ACC car-following model, on the other hand, is designed to describe the motion state of a vehicle while following the lead vehicle. In the ACC model, vehicle speed, position, and acceleration are the fundamental state variables. The acceleration and headway distance are adjusted through control algorithms to achieve following control. The ACC model assumes that a vehicle adjusts its speed and headway distance based on the speed and position of the preceding vehicle. Vehicle acceleration in the ACC model is calculated based on the current speed, the speed and distance of the preceding vehicle, and other parameters.

In emergency braking scenarios, drivers typically override system controls to avoid collisions [22], and car-following models must explicitly account for such collision avoidance behaviors [23]. In these scenarios, vehicles determine whether to perform emergency braking or lane changes based on the braking intensity of the lead vehicle and their own braking capabilities. During emergency braking, vehicles minimize their relative speed and position to reduce the risk of collision. When vehicles decelerate or accelerate at intersections, CACC may degrade to ACC, with a desired headway distance $\tau_{A}$ and a headway distance error $e^{\prime }$, typically set to specific values $e^{\prime }=0.1\text{m}$ [24]. The minimum headway distance $x_{cA}$ between an ACC vehicle and its lead vehicle during start-up time t0 is given by Equation (11):


$$x_{cA}=v_{s}\tau_{A}+x_{\Delta }+x_{m}+{e^{\prime }}_{s}+\frac{a_{s}\cdot t_{0}^{2}}{6}$$ (11)

The ACC model offers advantages in adaptive control of vehicles with respect to their lead vehicles, effectively improving driving comfort and safety. Additionally, the ACC model reduces driver fatigue and distraction, making driving more relaxed and enjoyable.

Unlike the IDM used by HDVs, which primarily focuses on headway distance s(t) and speed difference  Δ v(t), the CACC model further incorporates the acceleration of the lead vehicle. To this end, this paper explicitly includes key variables $X_{t}^{\left(c\right)}$ such as  Δ v and  Δ a in the state sub-vector of CAVs, as shown below:


$$X_{t}^{\left(c\right)}=\left[\begin{array}{ccccccc} \;\;\;\Delta \;\;\;x_{t,1}^{\left(c\right)}, & \;\;\;\Delta \;\;\;v_{t,1}^{\left(c\right)}, & \;\;\;\Delta \;\;\;a_{t,1}^{\left(c\right)}, & \ldots , & \;\;\;\Delta \;\;\;x_{t,i}^{\left(c\right)}, & \;\;\;\Delta \;\;\;v_{t,i}^{\left(c\right)}, & \;\;\;\Delta \;\;\;a_{t,i}^{\left(c\right)} \end{array}\right]$$ (12)

Where $\;\;\;\Delta \;\;\;x_{t,i}^{\left(c\right)}$ represents the distance deviation between the CAV and its lead vehicle in lane *i*; $\;\;\;\Delta \;\;\;v_{t,i}^{\left(c\right)}$ represents the speed difference of the CAV in lane *i*; $\;\;\;\Delta \;\;\;a_{t,i}^{\left(c\right)}$ represents the acceleration difference of the CAV in lane *i*.

When considering pedestrian crossing demands, it is necessary to reflect both pedestrian waiting and queuing conditions and the interactions between pedestrians and vehicles in terms of potential conflicts or arrival needs. Therefore, the pedestrian state at time *t* is defined as:


$$S_{t}^{\left(p\right)}=\left\{W_{t},N_{t},C_{t}\right\}$$ (13)

Where $W_{t}$, $N_{t}$, and $C_{t}$ represent pedestrian queuing/waiting information, arrival/demand information, and conflict/safety information, respectively.


$$S^{l}(t)=\{\phi (t),G_{r}(t),G_{p}(t\}$$ (14)

Where ϕ(t) denotes the currently active signal phase; $G_{r}(t)$ represents the remaining green light duration; $G_{p}(t)$ denotes the duration of the pedestrian-exclusive phase.

### Action function

**Fig 2:**
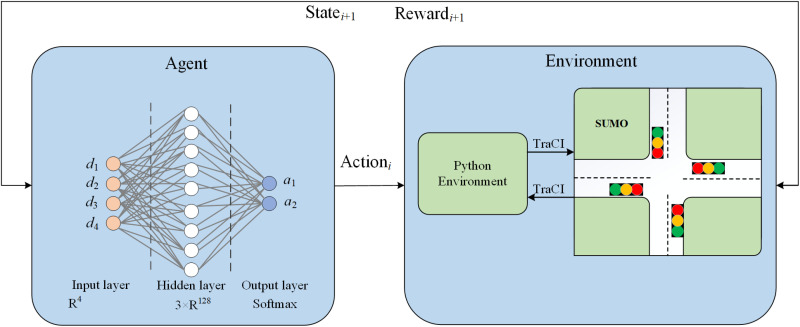
Deep reinforcement learning process.

In Deep Reinforcement Learning (DRL) and other adaptive optimization frameworks, the action function determines the control decisions that the signal controller should make at time *t* based on the current state. This paper previously defined state vectors to distinguish between HDV and CAV car-following models, as well as to capture the queuing and waiting information of pedestrians. Leveraging this information, the signal controller can dynamically adjust green light duration increments, phase switching timings, and pedestrian phase activations to optimize vehicle throughput while ensuring pedestrian safety. As illustrated in Fig 2, the figure depicts the process of reinforcement learning in this context.

Suppose an intersection has Ω vehicular phases (excluding pedestrian-vehicular shared phases). At time *t*, the agent needs to decide whether and how to adjust the green light duration increment or decrement for each vehicular phase. This can be defined as:


$$\;\;\;\Delta \;\;\;G_{t}=\left[\begin{array}{c} \;\;\;\Delta \;\;\;G_{t,1},\;\;\;\Delta \;\;\;G_{t,2},\ldots ,\;\;\;\Delta \;\;\;G_{t,\;\;\;\Omega \;\;\;} \end{array}\right]$$ (15)

Where $\;\;\;\Delta \;\;\;G_{t,\omega }$ represents the green light duration increment for vehicular phase ω in the next stage ($\;\;\;\Delta \;\;\;G_{t,\omega }$ > 0 extends the green light, $\;\;\;\Delta \;\;\;G_{t,\omega }$ < 0 shortens it, and $\;\;\;\Delta \;\;\;G_{t,\omega }$ = 0 keeps it unchanged).

In practical applications, the minimum green light duration $G_{\omega }^{\min}$ and maximum green light duration $G_{\omega }^{\max}$ must typically be satisfied, ensuring the following constraint:


$$G_{\omega }^{\min}\leq G_{\omega }^{\text{cur}}+\;\;\;\Delta \;\;\;G_{t,\omega }\leq G_{\omega }^{\text{max}}$$ (16)

The signal controller can extend the green light duration before it ends, based on the CAV proportion and estimated arrival times, to prevent platoons from stopping. Similarly, when an HDV queue accumulates and the dissipation wave lags significantly, the green light duration can be modestly increased in the next cycle to ensure queue dissipation.

In addition to incrementally fine-tuning the green light duration for individual phases, it is also necessary to make phase switching decisions, particularly in the interaction between pedestrian phases and vehicular phases, where extra caution is required. To this end, the phase-switching action can be defined as:


$$\psi_{t}\in \{1,2,\ldots ,\;\;\;\Omega \;\;\;,{\;\;\;\Omega \;\;\;}_{p}\}$$ (17)

Where $\psi_{t}$ represents the index of the phase to be executed in the next stage; {1,…, Ω } represents the vehicular phase indices, and ${\;\;\;\Omega \;\;\;}_{p}$ represents the pedestrian-exclusive phase.

For unsaturated traffic conditions, strict phase order may not be necessary, allowing $\psi_{t}$ direct jumps from the current phase to any other phase. However, in saturated or oversaturated conditions, fixed phase order constraints must be considered. In such cases, $\psi_{t}$ is further restricted to the index of the current phase ±1, with an additional Boolean variable indicating whether the pedestrian phase should be activated.

To address the heterogeneity between macroscopic and microscopic features, all input features were normalized using Z-score standardization to ensure consistent numerical ranges. Additionally, a weighting mechanism was applied to balance the contributions of macroscopic and microscopic indicators in the state vector, preventing any single feature type from dominating the learning process. These steps ensure the neural network effectively processes mixed-scale inputs while maintaining training stability.

### Reward function

In intersection signal control involving HDVs, CAVs, and pedestrians, it is essential to balance multiple objectives, including vehicle traffic efficiency (accounting for differences between HDVs and CAVs to minimize queues and delays), pedestrian traffic efficiency (avoiding excessive waiting and queuing), safety, and phase-switching stability (reducing vehicle-pedestrian conflict risks and the negative impact of frequent signal changes). A well-balanced reward design ensures that no stakeholder’s interest is excessively compromised or disproportionately prioritized. To achieve these objectives, this paper designs the immediate reward $R_{t}$ as a weighted combination of multiple sub-components, with penalty terms introduced for specific indicators to measure system performance comprehensively.


$$R_{t}=\left(R_{t}^{\left(\text{v}\right)},R_{t}^{\left(\text{p}\right)},R_{t}^{\left(\text{s}\right)},R_{t}^{\left(c\right)}\right)$$ (18)

Where $R_{t}^{\left(\text{v}\right)}$ quantifies vehicle traffic efficiency; $R_{t}^{\left(\text{p}\right)}$ quantifies pedestrian traffic efficiency; $R_{t}^{\left(\text{s}\right)}$ reflects safety and stability in phase switching; $R_{t}^{\left(c\right)}$ penalizes frequent phase switches to suppress excessive signal changes.

Considering the differences between HDVs and CAVs in mixed traffic flows, let $Q_{t}^{\left(h\right)}=\underset{M}{\overset{i=1}{\sum }}\;Q_{t,i}^{\left(h\right)}$ denote the queue size of HDVs at the intersection (vehicles) and $Q_{t}^{\left(c\right)}$ denote the total queue size of CAVs. Let $D_{t}^{\left(h\right)}$ and $D_{t}^{\left(c\right)}$ represent the average delay per vehicle for HDVs and CAVs (in seconds), respectively. The relationship between queue size Qt and average delay Dt can be expressed as [25]:


$$Qt=\frac{C_{t}T_{g}T_{t}}{4T_{C}}\left(\left(S_{t}-1\right)+\sqrt{{\left(S_{t}-1\right)}^{2}+\frac{12T_{C}(S_{t}-S_{0})}{C_{t}T_{t}T_{g}}}\right)$$ (19)

$$D_{t}=\frac{X_{t}T_{C}\cdot {\left(1-T_{g}/T_{C}\;\right)}^{2}}{2\cdot (1-X_{t}/C_{a,t}\;)}+S_{t}Q_{t}$$ (20)

Where: $X_{t}$ is the traffic flow rate on the segment; $C_{t}$ is the saturation flow rate of the segment; $S_{t}$ is the saturation level, defined as ${X_{t}/C\;}_{t}$; $T_{C}$ is the intersection phase cycle; $T_{g}$ is the green light phase duration; $T_{t}$ is the flow period, typically measured in hours; $S_{0}$ is the basic saturation of the road section.

To encourage the minimization of queue lengths and delays, we define the following negative measures:


$$R_{t}^{\left(\text{v},\text{HDV}\right)}=-\theta_{1}D_{t}^{\left(h\right)}$$ (21)

$$R_{t}^{\left(\text{v},\text{CAV}\right)}=-\theta_{2}D_{t}^{\left(c\right)}$$ (22)

The combined vehicle efficiency sub-reward is then defined as:


$$R_{t}^{\left(\text{v}\right)}=R_{t}^{\left(\text{v},\text{HDV}\right)}+R_{t}^{\left(\text{v},\text{CAV}\right)}$$ (23)

The negative sign ensures that greater queues and delays result in lower rewards, driving the algorithm to minimize these factors.

In pedestrian crossing scenarios, prolonged waiting times or surging queues often lead to impatience and risky crossing behavior, disrupting traffic order and safety. According to queueing theory and pedestrian behavioral models (e.g., the Social Force Model), pedestrian satisfaction decreases exponentially with waiting time, and queue lengths tend to accumulate non-linearly in high-flow scenarios. The pedestrian traffic efficiency sub-reward should reflect the relationship where larger waiting times and queue sizes result in lower rewards while preventing extreme cases.

Let $W_{t}^{\left(p\right)}$ represent the average waiting time of pedestrians across the intersection at time *t*. The pedestrian crossing reward function is defined as:


$$W_{t}^{\left(p\right)}=-\eta_{1}\cdot W_{t}-\eta_{2}\cdot \;\;\;\Theta \;\;\;(W_{t}-W_{th})$$ (24)

Where $\eta_{1}$, $\eta_{2}$ ≥0 are weight coefficients;  Θ (·) is the Heaviside step function, which equals 1 when $W_{t}^{\left(p\right)}>W_{\text{th}}$ (indicating pedestrian waiting time exceeds the threshold) and 0 otherwise, imposing an additional penalty for prolonged waiting.

The waiting time of pedestrians is measured on an individual basis, while the waiting time of vehicles depends on the passenger capacity. Therefore, multiple optimization objectives are weighted and summed up [11].

In mixed traffic and pedestrian scenarios, safety conflicts are often measured using near-miss indicators such as Time-to-Collision (TTC). In this paper, a vehicle-pedestrian or vehicle-vehicle conflict is considered a potential collision if the TTC falls below a certain threshold. A lower TTC frequency or severity indicates higher safety levels.

Let the position and velocity of the *i*-th vehicle and *p*-th pedestrian (or another vehicle) be $x_{i}(t)$, $v_{i}(t)$ and $x_{p}(t)$, $v_{p}(t)$ respectively. The Time-to-Collision (TTC) is defined as the minimum time required for the relative distance between them to reduce to zero starting from the current moment. Let: $x_{\text{rel}}(t)=x_{i}(t)-x_{p}(t)$, $v_{\text{rel}}(t)=v_{i}(t)-v_{p}(t)$.

If $v_{\text{rel}}(t)$ points toward and approaches $x_{\text{rel}}(t)$, then under simplified conditions (assuming non-zero relative velocity), the TTC can be expressed as:


$$\text{TT}{\text{C}}_{i,p}(t)=\frac{-x_{\text{rel}}(tcdotv_{\text{rel}}(t)}{∥v_{\text{rel}}(t){∥}^{2}}$$ (25)

If this condition is not satisfied, or the denominator equals 0 (e.g., identical speeds or zero relative velocity), TTC is defined as $\text{TT}{\text{C}}_{i,p}(t)=+\infty$, indicating no collision risk or that TTC is undefined.

Let $C_{t}$ represent the total number of detected conflicts (vehicle-pedestrian or vehicle-vehicle) at time *t*, and let $S_{t}^{\left(\text{c,s}\right)}$ denote the cumula*t*ive severity of these conflicts. To impose a negative penalty on safety-related conflicts within the reward function, the following is defined:


$$R_{t}^{\left(\text{s}\right)}=-\left(\xi_{1}C_{t}+\xi_{2}S_{t}^{\left(\text{c,s}\right)}\right)$$ (26)

Where: $\xi_{1}$, $\xi_{\text{2}}$ ≥0 are weights; When assessing only the number of conflicts, $\xi_{\text{2}}$ =0; When emphasizing conflict severity (e.g., minor vs. severe), $\xi_{\text{2}}$ can be assigned a higher weight, with additional penalties for severe conflicts.


$$S_{t}^{\left(c,s\right)}=\underset{C_{t}}{\overset{i=1}{\sum }}\;\frac{1}{TTC_{i}}$$ (27)

Where: $C_{t}$ represents the total number of detected conflict events at time *t*; $TTC_{i}$ represents the Time-to-Collision of *t*he *i*-th conflict event (in seconds).

The logic behind this formula is as follows: when $TTC_{i}$ is small, the risk of collision is higher, thus the value of 1/ $1/TTC_{i}\;$ is larger, indicating higher severity. Conversely, when $TTC_{i}$ is large, the collision risk is lower, and the value of $1/TTC_{i}\;$ becomes smaller, indicating lower severity.Additionally, we introduced an extra penalty term $\xi_{2}S_{t}^{\left(\text{c,s}\right)}$ in the reward function to further penalize high-severity conflict events. This ensures that the algorithm actively avoids low-TTC events during the training process.

Frequent phase switching can cause uncertainty for drivers and pedestrians, increase vehicle start-up delays, and weaken traffic flow stability. It may also lead to situations where pedestrian phases are repeatedly opened and closed, resulting in “false green lights” or “insufficient green duration,” which can tempt pedestrians to violate traffic rules. According to signal control theory, maintaining a moderate level of signal stability can mitigate the negative effects of frequent light changes on vehicle platoons.

Let $Y_{t}^{\left(c\right)}$ represent the number of phase switches (or the difference between actual switches and allowed limits) during decision period *t*. The penalty function for phase switching is defined as:


$$R_{t}^{\left(c\right)}=-\delta Y_{t}^{\left(c\right)}$$ (28)

Where δ ≥0 is the penalty coefficient. The higher the number of phase switches, the greater the penalty.

## Neural network architecture

### Main neural network design

**Fig 3:**
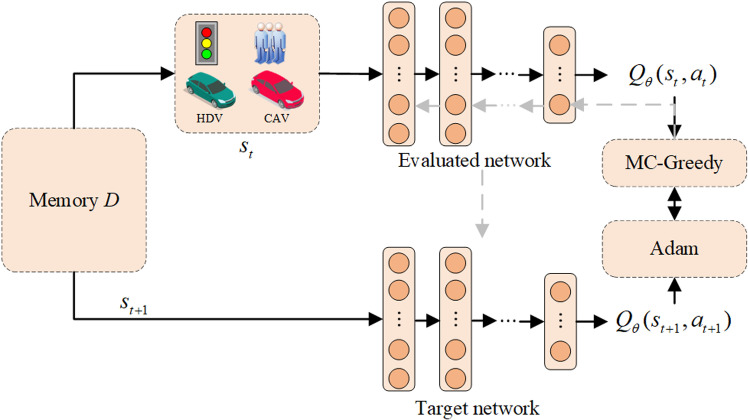
EXP-DDQN algorithm framework.

As shown in Fig 3, the EXP-DDQN algorithm is used to design the main neural network architecture in intelligent traffic signal control. The core components include the evaluation network, target network, and MC-Greedy strategy. This architecture aims to address the overestimation issue of Q-values in traditional Q-learning algorithms. By incorporating the MC-Greedy strategy and the target network, it effectively enhances the efficiency and accuracy of signal control.

The DDQN framework introduces both target and evaluation networks, which work together to improve the accuracy of Q-value estimations, thereby providing more reliable decision-making for traffic signal optimization. The input layer receives normalized state features (via Z-score standardization) to mitigate scale imbalances between heterogeneous data types. This design ensures that the network learns robust representations without bias toward specific feature scales.

#### Network architecture.

The main neural network adopts a deep neural network (DNN) to approximate the Q-function. We use a simple multilayer perceptron (MLP) structure, which includes an input layer, multiple hidden layers, and an output layer. The input layer captures traffic flow characteristics, and the output layer corresponds to the Q-value for each possible action.

Assuming the system is at time step *t* with state $s_{t}$ and takes action $a_{t}$, the output of the main network, $Q_{\theta }(s_{t},a_{t})$, represents the Q-value, which estimates the expected return of taking action $a_{t}$ in state $s_{t}$. The network adjusts its parameters θ through training to minimize prediction errors and improve Q-value accuracy.

The network’s input includes state features such as traffic flow, queue length, and pedestrian status, while the output represents the Q-value for each action. The Q-value for a specific action is calculated as:


$$Q_{\theta }(s_{t},a_{t})=\text{MLP}(s_{t};\theta )$$ (29)

Where θ represents the network’s weight parameters, $s_{t}$ denotes the state, and $a_{t}$ refers to the selected action.

#### Double network framework.

The DDQN framework utilizes both the main network and the target network to address the overestimation problem of Q-values in the DQN algorithm. The main and target networks share the same architecture, with the only difference being that the target network’s parameters are updated periodically by copying the parameters of the main network, rather than being updated with every training step.

The main network, $Q_{\theta }(s_{t},a_{t})$ is used to select the optimal action in the current state, while the target network, $Q_{\theta^{-}}(s_{t},a_{t})$ calculates the Q-value for that action. To reduce overestimation bias, the target Q-value is computed as:


$$y_{t}=r_{t}+\gamma \cdot Q_{\theta^{-}}(s_{t+1},\mathrm{\backslash arg}\underset{a^{\prime }}{\max}\;Q_{\theta }(s_{t+1},a^{\prime }))$$ (30)

Where $r_{t}$ is the immediate reward; γ is the discount factor; $Q_{\theta }(s_{t+1},a^{\prime })$ represents the Q-value output of the target network; and $argmaxQ_{0}(s_{t+1},a^{\prime })$ denotes the optimal action selected by the main network.

The parameters θ of the main network are periodically copied to the target network’s parameters $\theta^{-}$, ensuring the target network provides a stable Q-value for training and mitigates the instability caused by constant updates during training.

#### Activation function and optimizer.

Each layer of the main network employs the ReLU activation function. The ReLU activation function is widely used in deep learning due to its ability to reduce vanishing gradient problems and improve the network’s convergence efficiency. The ReLU function is defined as:


ReLU(x)=max(0,x) (31)

This means that for any input x, the output is x if x >0, and 0 otherwise.

To optimize the network, the Adam optimizer is used. This optimizer automatically adjusts the learning rate for each parameter based on the estimates of the first and second moments of the gradients, enhancing training speed and stability. The Adam optimization algorithm is expressed as:


$$\theta_{t+1}=\theta_{t}-\alpha \cdot \frac{{\hat{m}}_{t}}{\sqrt{{\hat{v}}_{t}}+\epsilon }$$ (32)

Where α is the learning rate, ${\hat{m}}_{t}$ is the mean estimate of the gradient, ${\hat{\nu }}_{t}$ is the mean estimate of the square of the gradient, and ϵ is a small constant to prevent zero division errors.

#### Loss function and Q-Value update.

The loss function is the core of neural network training, aiming to minimize the prediction error between the estimated Q-value and the target Q-value. The loss function is defined as:


$$L(\theta )={𝔼}_{(s,a,r,s^{\prime }simD}\left[{\left(y_{t}-Q_{\theta }(s_{t},a_{t})\right)}^{2}\right]$$ (33)

Where D is the replay buffer, containing multiple historical state-action-reward-next state tuples. The objective of the loss function is to minimize the mean squared error (MSE) between the predicted Q-value $Q_{\theta }(s_{t},a_{t})$ and the target Q-value $y_{t}$.

#### Exploration and exploitation strategy.

In reinforcement learning, the agent needs to balance exploration (discovering unknown actions in the state space) and exploitation (choosing the optimal action based on current knowledge to maximize the expected return). The MC-Greedy strategy is commonly used to achieve this balance, combining the Monte Carlo method with the epsilon-greedy approach.

In the MC-Greedy strategy, the agent calculates the cumulative return of each action based on historical experiences stored in the replay buffer. By comparing the returns of different actions, the agent tends to select the action with the highest return. However, to avoid falling into local optima, the MC-Greedy strategy maintains a certain level of randomness. Specifically, even if an action has a higher return, the agent still has a small probability of selecting other actions to ensure exploration diversity, thus improving learning efficiency and preventing premature convergence.

Assuming a discrete action space with |A| available actions, where |A| represents the number of actions, the MC-Greedy strategy is defined as:


$$\pi (a|s)=\{\frac{1-\frac{\varepsilon }{\left|A(s)\right|}(|A(s)|-1),\;if\;a=a^{*}}{\frac{\varepsilon }{\left|A(s)\right|},\;if\;a\neq a^{*}}$$ (34)

Where a is the action chosen in state s; |A(s)| is the number of available actions in state s; ε is the exploration rate, representing the probability of selecting a non-optimal action randomly; $a^{*}$ is the optimal action, defined as $a^{*}=\mathrm{\backslash arg}\underset{a\in A(s)}{\max}\;Q(s,a)$, the action with the highest Q-value.

The core of the MC-Greedy strategy lies in continuously adjusting the action selection probabilities based on historical experiences, ensuring sufficient exploration of the environment while guaranteeing efficient exploitation of the known rewards.

### Target network design

In Double Deep Q-Network (DDQN), the role of the target network is to stabilize target calculations and mitigate the overestimation issue found in traditional Q-learning methods. While the target network shares the same structure as the main network, its parameters are not updated during each training step but are instead periodically synchronized with those of the main network.

The core function of the target network is to compute the target Q-value, which is used to update the Q-learning process. In traditional Q-learning, the target Q-value is calculated using the current Q-values, which often results in overestimation errors. The DDQN approach addresses this problem by using the target network to compute the target Q-value based on the action selected by the main network with the highest Q-value.

The target Q-value is calculated as follows:


$$y_{t}=r_{t}+\gamma \cdot Q_{\theta^{-}}\left(s_{t+1},\mathrm{\backslash arg}\underset{a^{\prime }}{\max}\;Q_{\theta }(s_{t+1},a^{\prime })\right)$$ (35)

Where $r_{t}$ is the reward at the current time step; γ is the discount factor; $Q_{\theta }(s_{t+1},a^{\prime })$ is the Q-value computed by the target network; $\mathrm{\backslash arg}\underset{a^{\prime }}{\max}\;Q_{\theta }(s_{t+1},a^{\prime })$ is the action with the highest Q-value selected by the main network based on the next state $s_{t+1}$.

In this formula, the target network provides the Q-value estimation required for the selected optimal action (the action with the highest Q-value). By employing this approach, DDQN effectively avoids the overestimation problem in Q-value calculations and ensures stability during the learning process.

To prevent frequent updates of the target Q-value from being influenced by changes in the main network parameters $\theta^{-}$, the parameters of the target network, are only updated at fixed intervals. After every *N* training steps, the parameters $\theta^{-}$ of the target network, are synchronized with the current parameters θ of the main network.

## Case study

### Selection of research location

**Fig 4:**
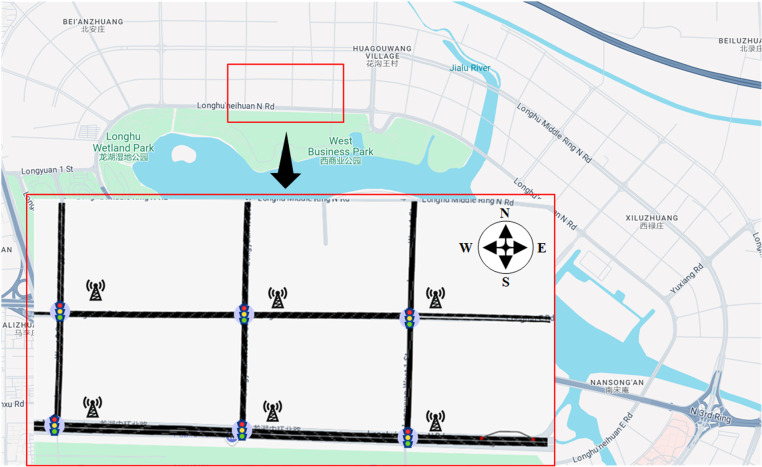
Simulated road network.

The selected simulation road network in this study is located in the autonomous driving test zone of Longhu Lake, Zhengzhou (as shown in Fig 4). The overall network configuration is as follows: the main road, Longhu Inner Ring North Road, is a dual six-lane road with partial sections containing auxiliary lanes, a main lane speed limit of 60 km/h, and auxiliary lanes limited to 40 km/h. The secondary road, a dual four-lane road, has a speed limit of 40 km/h.

In this study, to obtain accurate and realistic road network data, OpenStreetMap (OSM) data was used and processed through SUMO to import and transform the road network. This allowed reconstruction of the region’s road geometry, intersection locations, and basic traffic organization within the simulation platform. Considering the autonomous driving test zone’s characteristics and the abundant roadside detection equipment, the network can not only reflect the typical flow characteristics of HDVs but also simulate the driving efficiency improvements and road coordination properties brought by CAVs. Therefore, the selected road segment and its intersections were chosen for experiments to provide highly reliable data support for evaluating signal control strategies in mixed traffic flow scenarios.

**Fig 5:**
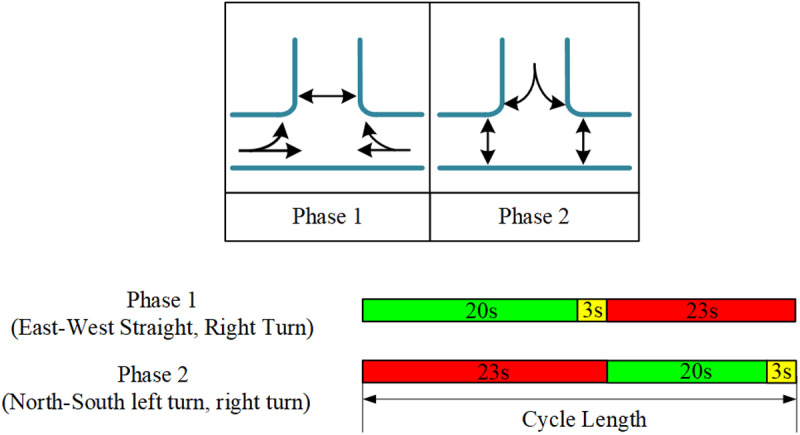
Phase timing and signal timing of four-way intersections.

**Fig 6:**
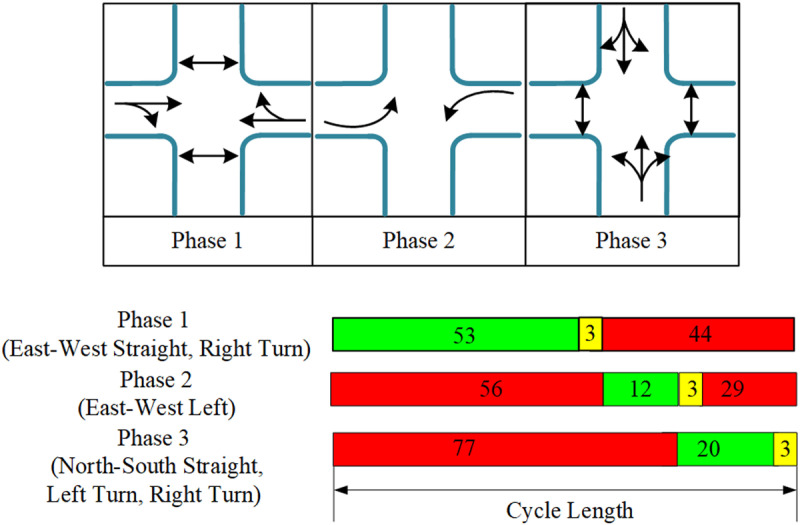
Phase timing and signal timing of three-way intersections.

In real-world road networks, the total number of phases (Ω) at an intersection depends on the number of intersecting roadways and their traffic flow characteristics. For a four-way intersection, the total number of phases consists of 2 vehicle phases and 2 pedestrian phases, resulting in a total of 4 phases. Similarly, for a three-way intersection (Fig 5), the total number of phases also includes 2 vehicle phases and 2 pedestrian phases, making a total of 4 phases. Specifically, the phase sequence for the four-way intersection includes: North-South through and left-turn, East-West through and left-turn, as well as pedestrian phases for both North-South and East-West crossings. For the three-way intersection (Fig 6), the phases consist of East-West through and left-turn, North-South left-turn, and pedestrian phases for both North-South and East-West crossings. In the diagrams, single arrows represent vehicle phases, while double arrows represent pedestrian phases. Each diagram also illustrates the green light duration ranges for each phase and their switching logic, ensuring that the signal control strategy can adapt to dynamic traffic demands while balancing the safety and efficiency of both pedestrians and vehicles.

The traffic volume distribution of the selected road network was obtained by investigating the traffic flow at various nodes and comparing it with the inflow and outflow data of adjacent road segments. Based on Monte Carlo methods for random allocation modeling, the concept of vehicle entry and exit points along the road was introduced. Simultaneously, 50% of the vehicles were randomly assigned as CAVs for flow distribution. Additionally, vehicles with a speed set to 0 were used to simulate the occurrence of emergency incidents. The neural network inherently adapts to heterogeneous input scales through nonlinear transformations, ensuring balanced feature contributions during training.

### Algorithm parameters and workflow

To address the balance between exploration and exploitation in signal control for mixed traffic flows, this study proposes the EXP-DDQN algorithm based on the MC-Greedy strategy. This algorithm dynamically adjusts the exploration rate and employs a replay buffer mechanism, effectively optimizing the learning process of the intelligent agent and enhancing the performance of signal control. Table 1 outlines the core steps of the algorithm.

**Table 1:** EXP-DDQN algorithm.

EXP-DDQN Algorithm
**Input:** State space S, action space A, learning rate α, discount factor γ, initial exploration rate $\varepsilon_{\max}$, minimum exploration rate $\varepsilon_{\min}$, exploration decay rate ρ, replay buffer size D, batch size B, target network update frequency C, number of episodes N, maximum time steps $T_{\max}$. **Output:** Optimized Q-network parameters θ
**Initialize**: Q-network Q(s,a;θ) and the target network $Q^{\prime }(s,a;\theta^{\prime })$, with $\theta^{\prime }\leftarrow \theta$; Replay buffer D; Set the initial exploration rate $\varepsilon \leftarrow \varepsilon_{\max}$.
**For each** episode *n* from 1 to *N*:
Reset the environment and observe the initial state $s_{0}$
For each time step *t* from 1 to $T_{\max}$: Action Selection: With probability ε, select a random action a Otherwise, select $a\leftarrow a=\mathrm{\backslash arg}\underset{a^{\prime }}{\max}\;Q(s,a^{\prime };\theta )$ Execute action a, observe reward r and next state $s^{\prime }$ Store $\left(s,a,r,s^{\prime }\right)$ in replay buffer D **If** D exceeds its size limit, remove the earliest transition Sample a minibatch of transitions $\left(s_{i},a_{i},r_{i},{s^{\prime }}_{i}\right)$ from D **For** each sample $\left(s_{i},a_{i},r_{i},{s^{\prime }}_{i}\right)$: calculation error δ: $\delta =r+\gamma Q^{\prime }(s^{\prime },\mathrm{\backslash arg}\underset{a^{\prime }}{\max}\;Q(s^{\prime },a^{\prime };\theta^{\prime });\theta^{\prime })-Q(s,a;\theta )$ Priority of updating experience $p_{t}=|\delta |+\epsilon$ Sample a small batch weighted by priority from experience pool *D* M: $P(\text{sample}proptop_{t}$ Calculate importance weight $w_{t}$: $w_{t}={\left(\frac{1}{N\cdot P(\text{sample})}\right)}^{\beta }$ If ${s^{\prime }}_{i}$ is terminal: $y_{i}\leftarrow r_{i}$ Else: $y_{i}\leftarrow r_{i}+\gamma Q^{\prime }(s_{i}^{\prime },\text{argma}{\text{x}}_{a^{\prime }}Q(s_{i}^{\prime },a^{\prime };\theta );\theta^{\prime })$ Perform gradient descent to update Q-network parameters θ: $L(\theta )={𝔼}_{(s,a,r,s^{\prime }simD}\left[{\left(y_{t}-Q_{\theta }(s_{t},a_{t})\right)}^{2}\right]$ **If** *t* mod C = 0: Update target network $\theta^{\prime }\leftarrow \theta$ b Update exploration rate: $\varepsilon \leftarrow \varepsilon_{\min}+(\varepsilon_{\max}-\varepsilon_{\min}cdote^{-\rho ⬝N}$ Update $s\leftarrow s^{\prime }$ If $s^{\prime }$ is terminal, break **End For**
**Output:** Optimized parameters θ

In the design and implementation of deep reinforcement learning algorithms, the rational setting of parameters directly impacts the model’s convergence speed and overall performance. To validate the effectiveness of the adaptive signal control strategy under mixed traffic flow and pedestrian crossing scenarios, this study refines key parameters of the Double Deep Q-Network (DDQN) algorithm. These refinements ensure that the algorithm simultaneously considers vehicle throughput efficiency and pedestrian waiting time, thereby improving the safety and stability of signal control. The specific parameter settings are detailed in Table 2.

**Table 2:** Parameter configuration for deep reinforcement learning algorithm.

Parameter Category	Parameter Name	Value or Description
Learning and Discount Parameters	Learning Rate	0.001
	Discount Factor	0.9
Exploration Strategy	Initial Exploration Rate	0.92
	Minimum Exploration Rate	0.01
	Exploration Decay Rate	20,000
Replay Buffer	Replay Buffer Capacity	100000
	Batch Size	10
Target Network	Parameter Update Frequency	40%
Reward Function Weights	Vehicle Throughput Efficiency	30%
	Pedestrian Throughput Efficiency	20%
	Safety	10%
	Signal Stability	1000
Training Settings	Number of Episodes	2000
	Time Steps per Episode	128
Network Architecture	Number of Hidden Layers	3*128
	Number of Neurons per Layer	0.9

## Results and discussion

### Training results

**Fig 7:**
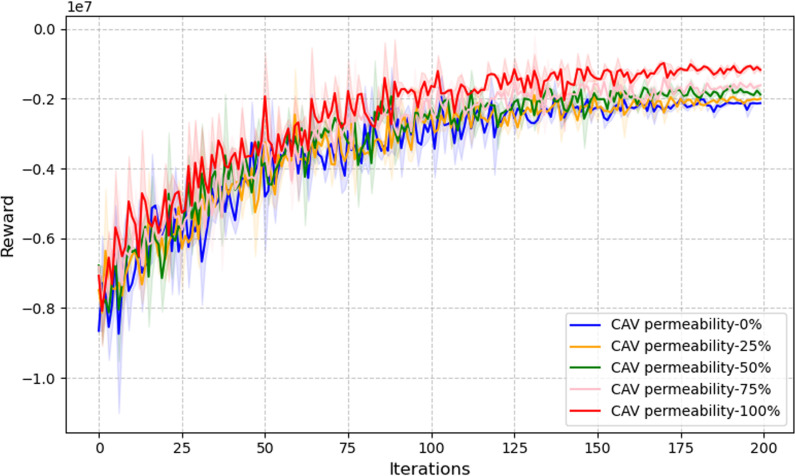
Cumulative reward variations at different CAV penetration rates.

As shown in Fig 5, when the CAV penetration rate is high, the system’s reward value initially rises rapidly, followed by stabilization during the learning process. Additionally, the reward value for high penetration rates is significantly greater than that for lower rates, indicating that increased CAV penetration leverages advantages such as higher vehicle speeds, reduced headways, and optimized signal timing, effectively improving traffic efficiency. Note that the model showed significant convergence by the 150th episode. Therefore, only the first 200 episodes are shown in Fig 7 for clarity.

In the figure, the curve for 100% CAV penetration consistently maintains the highest position throughout training, with smaller fluctuations and faster convergence compared to other curves. This demonstrates that the uniform behavior of CAVs allows the signal control strategy to align more effectively with an ideal scheduling plan. Conversely, the curve for 0% CAV penetration exhibits more noticeable initial oscillations and achieves the lowest final reward value, reflecting the higher randomness of HDVs. This randomness hinders the system’s adaptability, making signal control less responsive and slower in adjusting to changing conditions.

Moreover, the results highlight that in scenarios with intermediate penetration rates, integrating coordinated vehicle-road systems with adaptive signal control achieves a balance, yielding better training efficiency and higher reward levels.

### Test results

**Fig 8:**
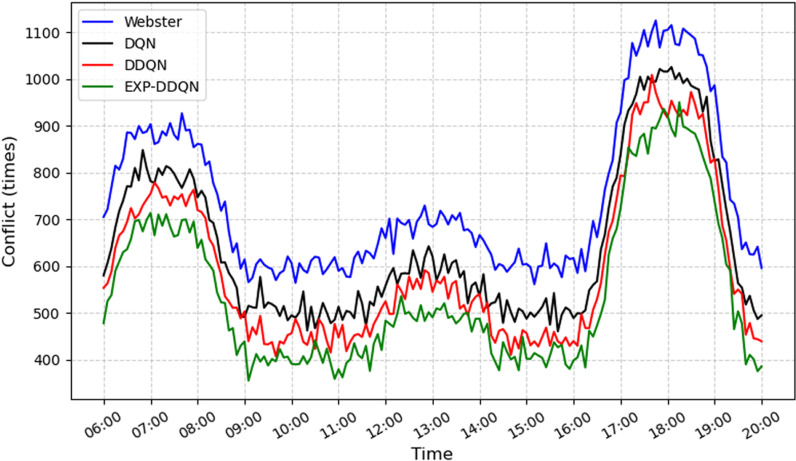
Comparison of conflict frequency across different algorithms.

**Fig 9:**
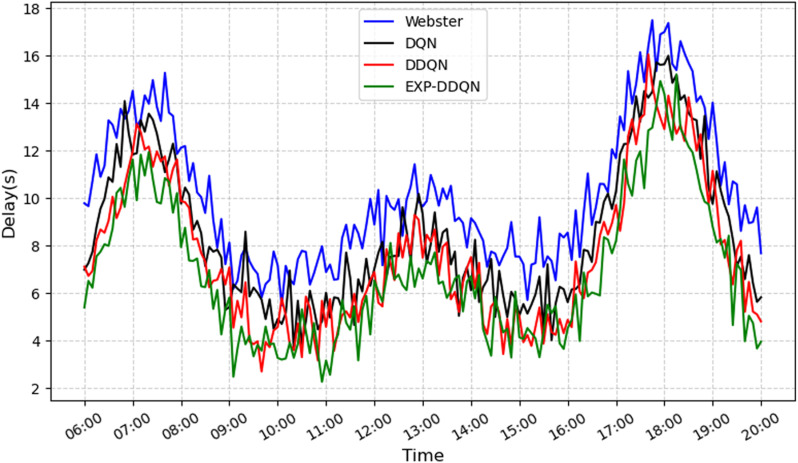
Comparison of average vehicle delay across different algorithms.

**Fig 10:**
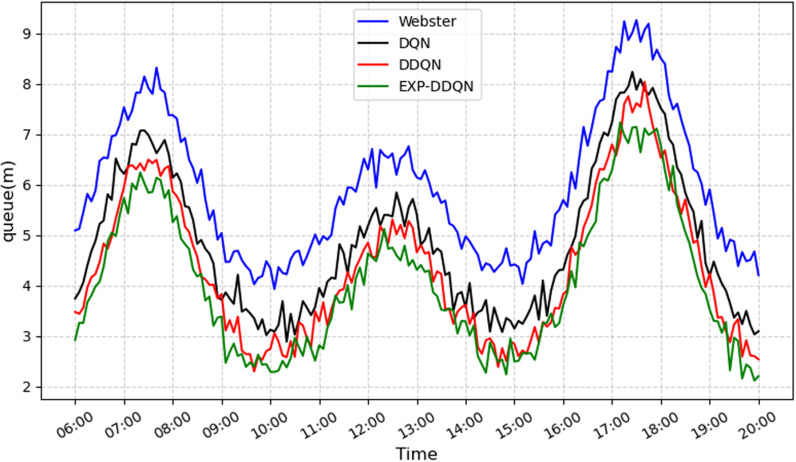
Comparison of queue length across different algorithms.

**Fig 11:**
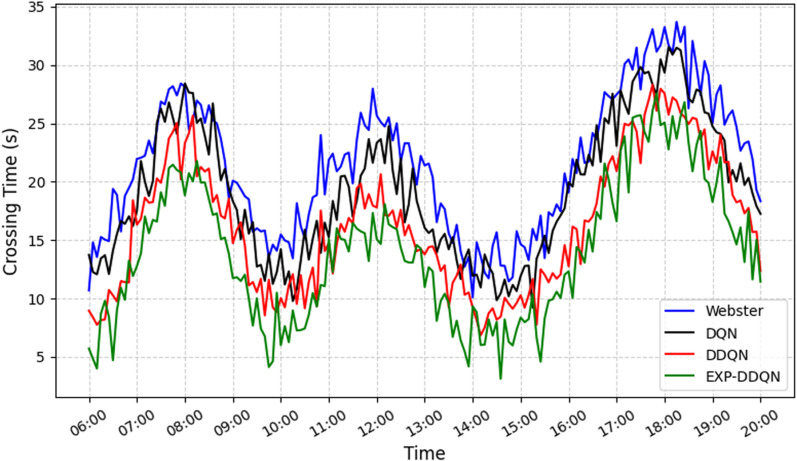
Comparison of pedestrian waiting time using different algorithms.

In this section, the proposed EXP-DDQN algorithm is compared comprehensively with three external strategies (Webster [26], DQN [27], and DDQN) to demonstrate its overall advantages under different traffic demand levels and various intersection environments [28]. Figs 8–11 present the queue length and delay metrics for the three strategies. A detailed comparison of the average daily results is provided in Table 3, showcasing pedestrian-vehicle conflict frequency, vehicle queue lengths, and average delays.

**Table 3:** Comparison of metrics across different methods.

Algorithm	Pedestrian-Vehicle Conflict Frequency (times)	Vehicle Queue (veh)	Average Delay (s)	Average pedestrian crossing wait times(s)
Webster	745	5.97	10.21	21.58
DQN	648	4.89	8.5	19.33
DDQN	596	4.38	7.64	16.24
EXP-DDQN	545	4.07	6.89	13.99

To better illustrate the relative performance of each method during different time periods or demand levels, the day is divided into four intervals: morning peak (7:00–9:00), midday peak (12:00–14:00), evening peak (17:00–19:00), and off-peak hours (remaining periods). For each interval, the average values of conflicts, queues, and delays are calculated.

From a daily time-period perspective, the Webster method exhibits significantly higher values for conflicts, queues, and delays during the morning peak (6:30–8:30) and evening peak (16:00–19:00) compared to reinforcement learning strategies. Conversely, DQN, DDQN, and EXP-DDQN are able to dynamically balance traffic demand and signal control allocation during high-flow periods through continuous learning and adaptive adjustments, maintaining lower levels of conflicts, queue sizes, and delays throughout most time periods. Notably, EXP-DDQN achieves the lowest metrics for conflicts, queues, and delays during peak periods among the four methods. This highlights the algorithm’s enhanced flexibility and precision in traffic management, enabled by its improved exploration-exploitation balance under a deep reinforcement learning framework, allowing it to better handle significant traffic fluctuations.

The results demonstrate that EXP-DDQN achieves the most significant improvements over Webster, DQN, and DDQN during peak-demand periods. Specifically, it reduces average hourly conflict frequency by 26.9%, 15.88%, and 8.6%, respectively; decreases average hourly queue length by 31.83%, 16.77%, and 7.08%; cuts down average hourly vehicle delay by 32.52%, 18.94%, and 9.82%; and shortens pedestrian crossing wait times by 35.17%, 27.63%, and 13.85%. These differences are particularly pronounced during peak traffic periods, indicating that reinforcement learning algorithms, especially EXP-DDQN, excel in traffic signal coordination and vehicle flow dissipation when traffic exceeds a certain saturation threshold. The algorithm’s ability to capture micro-level relationships between vehicle platoons and residual green time further reduces congestion and conflict risks at intersections.

Further analysis of spatiotemporal differences across intersections reveals that EXP-DDQN not only delivers the best overall network-wide performance but also maintains stability at most key intersections and road sections. While a few local intersections exhibit slightly higher delay metrics compared to some methods during specific time periods, this is likely due to the global signal scheduling strategy, which optimizes overall network performance at the expense of a few nodes [29].

Standard deviation and variance calculations indicate that EXP-DDQN and DDQN achieve more consistent performance, whereas Webster, DQN, and DDQN display greater fluctuations during peak periods, highlighting their relative instability.

These findings underscore that EXP-DDQN not only outperforms traditional fixed-time and earlier reinforcement learning methods in mitigating conflicts, enhancing safety, and reducing delays but also demonstrates strong adaptability and coordination across intersections. This highlights the significant potential and scalability of deep reinforcement learning in modern urban traffic signal control systems.

## Conclusions

By incorporating the micro-driving characteristics of CAVs and HDVs, such as speed and queue length, alongside pedestrian-related variables like waiting numbers and crossing frequency, this study provides a multidimensional representation of intersection traffic dynamics. This unified characterization enables reinforcement learning algorithms to effectively balance the needs of pedestrians and vehicles during training, significantly reducing vehicle conflicts and pedestrian crowding risks during peak hours or unexpected disruptions.

The proposed adaptive method leverages real-time traffic data from roadside and onboard sensors to dynamically adjust phase transitions and green light allocations. Compared to traditional fixed-time control strategies, it achieves superior queue dissipation and pedestrian crossing efficiency during fluctuating peak traffic periods. With an enhanced network structure and optimized training strategy, the method demonstrates faster learning convergence and robust performance across varying CAV penetration rates and pedestrian flow levels.

Experimental results validate the algorithm’s advantages: a 26.9% reduction in vehicle-pedestrian conflicts, a 32.52% decrease in traffic delays, and consistent scheduling stability even under extreme conditions where pedestrian and vehicle flows peak simultaneously. These outcomes highlight the method’s ability to maintain pedestrian safety while preserving traffic efficiency through dynamic intersection management.

The MC-Greedy exploration strategy in EXP-DDQN effectively balances exploration of novel control policies and exploitation of learned strategies, enabling precise signal regulation under traffic flow fluctuations. This adaptability addresses a critical limitation of traditional methods, which often overlook pedestrian impacts, while enhancing the granularity of traffic control through real-time adjustments to phase timing and pedestrian priority.

This study’s deep reinforcement learning-based adaptive signal control method significantly improves safety, efficiency, and robustness. Under varying traffic conditions, the EXP-DDQN method adapts effectively by balancing exploration and exploitation, demonstrating improved performance in both low and high CAV penetration scenarios. However, its performance may be limited in highly congested networks with extreme pedestrian flows, where further adjustments to the algorithm could be needed.

Future work could focus on integrating multi-point urban detection and V2X information-sharing technologies to address these challenges and further enhance system performance. Additionally, advanced pedestrian behavior prediction models can be developed to more accurately account for pedestrian impact. Further research could explore multi-intersection control strategies to optimize signal control in large-scale, complex road networks, thereby improving the efficiency and safety of urban traffic systems.

## Supporting information

S1 Dataset Supplementary data set description. (DOCX)
