# Supplementary material for: Intelligent connected adaptive signal control considering pedestrians based on the EXP-DDQN algorithm
Source: PLoS One. 2025 Jun 6;20(6):e0322945. doi: 10.1371/journal.pone.0322945 (PMC12143511; doi:10.1371/journal.pone.0322945)
Supplement: S1 Dataset — (DOCX) [file pone.0322945.s001.docx]

**Supplementary Data Set Description**

This dataset contains the pedestrian-vehicle conflict frequency, vehicle queue, average delay, and average pedestrian crossing wait times in this paper, aiming to support the experimental results and conclusions presented in the paper.

**Table 1. Pedestrian-vehicle conflict frequency and vehicle queue data.**

| **Time** | Pedestrian-Vehicle Conflict Frequency (times) | | | | Vehicle Queue (veh) | | | |
| --- | --- | --- | --- | --- | --- | --- | --- | --- |
|  | **Webster** | **DQN** | **DDQN** | **EXP-DDQN** | **Webster** | **DQN** | **DDQN** | **EXP-DDQN** |
| 06:00 | 706 | 581 | 554 | 478 | 5.09 | 3.74 | 3.48 | 2.92 |
| 06:05 | 722 | 607 | 564 | 526 | 5.12 | 3.87 | 3.44 | 3.26 |
| 06:10 | 768 | 639 | 589 | 538 | 5.45 | 4.06 | 3.56 | 3.26 |
| 06:15 | 815 | 683 | 639 | 590 | 5.82 | 4.4 | 3.96 | 3.67 |
| 06:20 | 807 | 718 | 666 | 611 | 5.67 | 4.68 | 4.16 | 3.8 |
| 06:25 | 830 | 741 | 675 | 630 | 5.88 | 4.88 | 4.23 | 3.98 |
| 06:30 | 886 | 771 | 695 | 637 | 6.47 | 5.22 | 4.46 | 4.07 |
| 06:35 | 885 | 770 | 725 | 658 | 6.54 | 5.29 | 4.83 | 4.37 |
| 06:40 | 872 | 810 | 702 | 696 | 6.52 | 5.81 | 4.73 | 4.87 |
| 06:45 | 900 | 784 | 711 | 699 | 6.96 | 5.7 | 4.97 | 5.05 |
| 06:50 | 885 | 849 | 730 | 675 | 6.98 | 6.52 | 5.33 | 4.98 |
| 06:55 | 888 | 810 | 743 | 699 | 7.2 | 6.31 | 5.65 | 5.41 |
| 07:00 | 904 | 782 | 755 | 714 | 7.53 | 6.21 | 5.95 | 5.74 |
| 07:05 | 861 | 778 | 779 | 666 | 7.28 | 6.35 | 6.36 | 5.43 |
| 07:10 | 865 | 810 | 767 | 711 | 7.47 | 6.81 | 6.39 | 6.02 |
| 07:15 | 889 | 796 | 747 | 687 | 7.82 | 6.79 | 6.3 | 5.9 |
| 07:20 | 880 | 814 | 750 | 711 | 7.82 | 7.07 | 6.42 | 6.24 |
| 07:25 | 906 | 809 | 730 | 685 | 8.14 | 7.08 | 6.28 | 6.03 |
| 07:30 | 882 | 798 | 749 | 664 | 7.92 | 6.99 | 6.5 | 5.84 |
| 07:35 | 871 | 782 | 743 | 667 | 7.8 | 6.81 | 6.42 | 5.86 |
| 07:40 | 927 | 768 | 754 | 699 | 8.32 | 6.62 | 6.49 | 6.14 |
| 07:45 | 891 | 786 | 729 | 700 | 7.89 | 6.75 | 6.17 | 6.09 |
| 07:50 | 892 | 808 | 751 | 672 | 7.83 | 6.89 | 6.32 | 5.73 |
| 07:55 | 855 | 787 | 764 | 696 | 7.38 | 6.61 | 6.37 | 5.89 |
| 08:00 | 862 | 748 | 720 | 639 | 7.38 | 6.14 | 5.86 | 5.25 |
| 08:05 | 860 | 762 | 716 | 657 | 7.31 | 6.22 | 5.77 | 5.38 |
| 08:10 | 816 | 747 | 703 | 615 | 6.84 | 6.05 | 5.61 | 4.93 |
| 08:15 | 824 | 699 | 659 | 604 | 6.92 | 5.57 | 5.17 | 4.82 |
| 08:20 | 778 | 693 | 645 | 591 | 6.5 | 5.55 | 5.06 | 4.73 |
| 08:25 | 755 | 662 | 611 | 544 | 6.33 | 5.3 | 4.79 | 4.31 |
| 08:30 | 719 | 608 | 583 | 523 | 6.03 | 4.83 | 4.57 | 4.18 |
| 08:35 | 738 | 609 | 536 | 522 | 6.3 | 4.9 | 4.18 | 4.23 |
| 08:40 | 674 | 586 | 525 | 463 | 5.71 | 4.73 | 4.12 | 3.7 |
| 08:45 | 630 | 573 | 511 | 468 | 5.3 | 4.63 | 4.01 | 3.78 |
| 08:50 | 649 | 554 | 512 | 410 | 5.49 | 4.44 | 4.02 | 3.2 |
| 08:55 | 595 | 492 | 489 | 430 | 4.91 | 3.78 | 3.75 | 3.36 |
| 09:00 | 615 | 492 | 504 | 439 | 5.04 | 3.71 | 3.83 | 3.39 |
| 09:05 | 566 | 516 | 440 | 356 | 4.47 | 3.86 | 3.11 | 2.46 |
| 09:10 | 576 | 513 | 470 | 386 | 4.47 | 3.74 | 3.31 | 2.68 |
| 09:15 | 605 | 511 | 455 | 412 | 4.67 | 3.63 | 3.07 | 2.85 |
| 09:20 | 615 | 577 | 494 | 396 | 4.69 | 4.21 | 3.38 | 2.6 |
| 09:25 | 604 | 512 | 434 | 408 | 4.5 | 3.48 | 2.71 | 2.64 |
| 09:30 | 598 | 523 | 433 | 388 | 4.38 | 3.53 | 2.64 | 2.38 |
| 09:35 | 594 | 519 | 438 | 402 | 4.3 | 3.45 | 2.64 | 2.47 |
| 09:40 | 570 | 513 | 408 | 397 | 4.02 | 3.35 | 2.3 | 2.39 |
| 09:45 | 586 | 494 | 439 | 423 | 4.15 | 3.13 | 2.59 | 2.63 |
| 09:50 | 591 | 515 | 435 | 405 | 4.19 | 3.33 | 2.53 | 2.43 |
| 09:55 | 621 | 485 | 453 | 407 | 4.48 | 3.02 | 2.7 | 2.44 |
| 10:00 | 607 | 495 | 457 | 392 | 4.34 | 3.12 | 2.74 | 2.29 |
| 10:05 | 565 | 490 | 488 | 390 | 3.93 | 3.08 | 3.06 | 2.28 |
| 10:10 | 606 | 502 | 469 | 391 | 4.36 | 3.22 | 2.89 | 2.31 |
| 10:15 | 592 | 546 | 438 | 408 | 4.25 | 3.69 | 2.61 | 2.5 |
| 10:20 | 586 | 463 | 432 | 392 | 4.22 | 2.88 | 2.58 | 2.37 |
| 10:25 | 612 | 514 | 460 | 406 | 4.52 | 3.44 | 2.9 | 2.56 |
| 10:30 | 621 | 468 | 424 | 442 | 4.65 | 3.03 | 2.58 | 2.96 |
| 10:35 | 619 | 491 | 487 | 417 | 4.69 | 3.31 | 3.27 | 2.78 |
| 10:40 | 583 | 522 | 474 | 393 | 4.4 | 3.69 | 3.21 | 2.61 |
| 10:45 | 594 | 501 | 441 | 424 | 4.58 | 3.56 | 2.95 | 2.98 |
| 10:50 | 607 | 478 | 416 | 392 | 4.79 | 3.41 | 2.78 | 2.74 |
| 10:55 | 620 | 486 | 477 | 359 | 5.01 | 3.57 | 3.49 | 2.51 |
| 11:00 | 590 | 514 | 448 | 380 | 4.82 | 3.95 | 3.29 | 2.81 |
| 11:05 | 596 | 486 | 475 | 363 | 4.98 | 3.77 | 3.66 | 2.74 |
| 11:10 | 578 | 505 | 418 | 393 | 4.91 | 4.07 | 3.21 | 3.16 |
| 11:15 | 577 | 502 | 439 | 401 | 5.01 | 4.15 | 3.53 | 3.35 |
| 11:20 | 618 | 489 | 452 | 436 | 5.53 | 4.14 | 3.77 | 3.8 |
| 11:25 | 631 | 547 | 455 | 411 | 5.76 | 4.82 | 3.9 | 3.66 |
| 11:30 | 607 | 521 | 449 | 404 | 5.6 | 4.64 | 3.93 | 3.67 |
| 11:35 | 633 | 473 | 476 | 430 | 5.94 | 4.24 | 4.27 | 4.01 |
| 11:40 | 628 | 524 | 449 | 376 | 5.94 | 4.8 | 4.05 | 3.52 |
| 11:45 | 617 | 516 | 477 | 434 | 5.86 | 4.75 | 4.36 | 4.13 |
| 11:50 | 647 | 557 | 492 | 455 | 6.17 | 5.17 | 4.53 | 4.36 |
| 11:55 | 681 | 535 | 511 | 421 | 6.52 | 4.95 | 4.71 | 4.01 |
| 12:00 | 660 | 559 | 525 | 484 | 6.3 | 5.18 | 4.85 | 4.63 |
| 12:05 | 702 | 581 | 498 | 477 | 6.71 | 5.39 | 4.57 | 4.56 |
| 12:10 | 627 | 596 | 498 | 471 | 5.94 | 5.54 | 4.56 | 4.48 |
| 12:15 | 702 | 562 | 561 | 498 | 6.69 | 5.18 | 5.18 | 4.75 |
| 12:20 | 693 | 584 | 548 | 536 | 6.58 | 5.4 | 5.03 | 5.12 |
| 12:25 | 689 | 585 | 530 | 498 | 6.53 | 5.4 | 4.84 | 4.73 |
| 12:30 | 699 | 584 | 578 | 502 | 6.62 | 5.37 | 5.31 | 4.75 |
| 12:35 | 659 | 634 | 551 | 490 | 6.19 | 5.84 | 5.01 | 4.6 |
| 12:40 | 695 | 608 | 573 | 483 | 6.52 | 5.55 | 5.2 | 4.5 |
| 12:45 | 707 | 575 | 552 | 517 | 6.59 | 5.17 | 4.94 | 4.79 |
| 12:50 | 730 | 619 | 592 | 483 | 6.76 | 5.55 | 5.28 | 4.39 |
| 12:55 | 690 | 643 | 586 | 502 | 6.29 | 5.72 | 5.14 | 4.51 |
| 13:00 | 684 | 621 | 546 | 491 | 6.14 | 5.41 | 4.66 | 4.31 |
| 13:05 | 690 | 570 | 570 | 510 | 6.11 | 4.81 | 4.8 | 4.4 |
| 13:10 | 719 | 591 | 563 | 507 | 6.29 | 4.91 | 4.63 | 4.27 |
| 13:15 | 707 | 626 | 578 | 521 | 6.05 | 5.14 | 4.66 | 4.29 |
| 13:20 | 689 | 586 | 531 | 490 | 5.76 | 4.63 | 4.07 | 3.86 |
| 13:25 | 709 | 608 | 563 | 494 | 5.85 | 4.73 | 4.28 | 3.79 |
| 13:30 | 699 | 613 | 569 | 478 | 5.64 | 4.67 | 4.23 | 3.52 |
| 13:35 | 714 | 576 | 510 | 486 | 5.68 | 4.21 | 3.54 | 3.5 |
| 13:40 | 677 | 590 | 517 | 499 | 5.23 | 4.26 | 3.53 | 3.54 |
| 13:45 | 679 | 521 | 495 | 501 | 5.18 | 3.5 | 3.24 | 3.5 |
| 13:50 | 671 | 558 | 524 | 461 | 5.05 | 3.83 | 3.48 | 3.05 |
| 13:55 | 641 | 566 | 535 | 488 | 4.73 | 3.87 | 3.56 | 3.29 |
| 14:00 | 667 | 536 | 541 | 488 | 4.98 | 3.57 | 3.62 | 3.29 |
| 14:05 | 656 | 583 | 502 | 459 | 4.87 | 4.05 | 3.24 | 3 |
| 14:10 | 640 | 511 | 522 | 477 | 4.73 | 3.35 | 3.46 | 3.21 |
| 14:15 | 625 | 521 | 452 | 414 | 4.61 | 3.47 | 2.78 | 2.6 |
| 14:20 | 592 | 523 | 437 | 396 | 4.3 | 3.51 | 2.64 | 2.44 |
| 14:25 | 605 | 542 | 462 | 378 | 4.44 | 3.71 | 2.91 | 2.27 |
| 14:30 | 601 | 479 | 466 | 438 | 4.4 | 3.08 | 2.95 | 2.87 |
| 14:35 | 588 | 528 | 454 | 417 | 4.27 | 3.56 | 2.82 | 2.66 |
| 14:40 | 599 | 502 | 411 | 401 | 4.36 | 3.3 | 2.38 | 2.49 |
| 14:45 | 609 | 481 | 449 | 407 | 4.45 | 3.08 | 2.76 | 2.53 |
| 14:50 | 638 | 510 | 424 | 378 | 4.74 | 3.35 | 2.5 | 2.24 |
| 14:55 | 604 | 504 | 464 | 449 | 4.39 | 3.3 | 2.89 | 2.95 |
| 15:00 | 605 | 488 | 457 | 403 | 4.42 | 3.15 | 2.84 | 2.49 |
| 15:05 | 599 | 501 | 431 | 402 | 4.37 | 3.3 | 2.6 | 2.51 |
| 15:10 | 562 | 492 | 440 | 415 | 4.03 | 3.24 | 2.71 | 2.66 |
| 15:15 | 599 | 502 | 429 | 410 | 4.45 | 3.38 | 2.65 | 2.65 |
| 15:20 | 601 | 513 | 449 | 404 | 4.53 | 3.55 | 2.9 | 2.66 |
| 15:25 | 649 | 532 | 469 | 384 | 5.08 | 3.8 | 3.18 | 2.53 |
| 15:30 | 596 | 475 | 430 | 409 | 4.63 | 3.32 | 2.87 | 2.86 |
| 15:35 | 606 | 543 | 460 | 438 | 4.83 | 4.1 | 3.27 | 3.25 |
| 15:40 | 599 | 461 | 439 | 427 | 4.88 | 3.4 | 3.18 | 3.26 |
| 15:45 | 577 | 497 | 434 | 432 | 4.79 | 3.89 | 3.27 | 3.44 |
| 15:50 | 623 | 512 | 448 | 390 | 5.41 | 4.2 | 3.56 | 3.18 |
| 15:55 | 616 | 506 | 430 | 381 | 5.5 | 4.31 | 3.54 | 3.25 |
| 16:00 | 617 | 489 | 441 | 399 | 5.7 | 4.31 | 3.83 | 3.61 |
| 16:05 | 586 | 500 | 430 | 405 | 5.56 | 4.6 | 3.9 | 3.85 |
| 16:10 | 637 | 499 | 498 | 431 | 6.25 | 4.77 | 4.76 | 4.28 |
| 16:15 | 590 | 506 | 468 | 384 | 5.92 | 4.98 | 4.61 | 3.96 |
| 16:20 | 644 | 549 | 468 | 462 | 6.57 | 5.52 | 4.71 | 4.85 |
| 16:25 | 697 | 560 | 507 | 450 | 7.14 | 5.68 | 5.15 | 4.77 |
| 16:30 | 662 | 568 | 530 | 473 | 6.77 | 5.73 | 5.35 | 4.98 |
| 16:35 | 707 | 637 | 564 | 498 | 7.12 | 6.32 | 5.59 | 5.13 |
| 16:40 | 764 | 668 | 624 | 529 | 7.52 | 6.46 | 6.02 | 5.27 |
| 16:45 | 799 | 725 | 674 | 626 | 7.66 | 6.82 | 6.31 | 6.02 |
| 16:50 | 827 | 771 | 697 | 659 | 7.7 | 7.03 | 6.3 | 6.12 |
| 16:55 | 907 | 789 | 744 | 680 | 8.25 | 6.97 | 6.52 | 6.08 |
| 17:00 | 929 | 839 | 794 | 724 | 8.24 | 7.24 | 6.8 | 6.29 |
| 17:05 | 998 | 904 | 793 | 782 | 8.74 | 7.69 | 6.58 | 6.67 |
| 17:10 | 1003 | 933 | 841 | 854 | 8.62 | 7.82 | 6.9 | 7.23 |
| 17:15 | 1078 | 946 | 924 | 841 | 9.24 | 7.83 | 7.6 | 6.98 |
| 17:20 | 1050 | 968 | 949 | 836 | 8.87 | 7.95 | 7.75 | 6.82 |
| 17:25 | 1073 | 1005 | 925 | 875 | 9.02 | 8.24 | 7.43 | 7.13 |
| 17:30 | 1105 | 977 | 950 | 884 | 9.26 | 7.88 | 7.62 | 7.15 |
| 17:35 | 1070 | 1006 | 951 | 841 | 8.83 | 8.09 | 7.54 | 6.64 |
| 17:40 | 1102 | 994 | 1009 | 897 | 9.07 | 7.88 | 8.04 | 7.11 |
| 17:45 | 1125 | 995 | 972 | 895 | 9.19 | 7.79 | 7.55 | 6.98 |
| 17:50 | 1068 | 1022 | 947 | 914 | 8.48 | 7.92 | 7.18 | 7.04 |
| 17:55 | 1104 | 1017 | 931 | 937 | 8.68 | 7.71 | 6.85 | 7.11 |
| 18:00 | 1105 | 1016 | 918 | 923 | 8.5 | 7.51 | 6.53 | 6.78 |
| 18:05 | 1116 | 1026 | 954 | 895 | 8.4 | 7.4 | 6.68 | 6.28 |
| 18:10 | 1075 | 1000 | 935 | 878 | 7.76 | 6.91 | 6.25 | 5.88 |
| 18:15 | 1073 | 1013 | 921 | 951 | 7.49 | 6.79 | 5.87 | 6.37 |
| 18:20 | 1108 | 992 | 935 | 899 | 7.6 | 6.34 | 5.77 | 5.61 |
| 18:25 | 1101 | 1001 | 923 | 895 | 7.29 | 6.2 | 5.42 | 5.34 |
| 18:30 | 1094 | 986 | 973 | 888 | 7.02 | 5.84 | 5.71 | 5.06 |
| 18:35 | 1087 | 982 | 947 | 884 | 6.77 | 5.62 | 5.28 | 4.84 |
| 18:40 | 1052 | 978 | 916 | 863 | 6.31 | 5.47 | 4.85 | 4.52 |
| 18:45 | 1051 | 930 | 926 | 835 | 6.25 | 4.94 | 4.89 | 4.18 |
| 18:50 | 1027 | 963 | 872 | 810 | 6.02 | 5.28 | 4.38 | 3.95 |
| 18:55 | 974 | 868 | 821 | 788 | 5.6 | 4.44 | 3.97 | 3.83 |
| 19:00 | 987 | 826 | 830 | 739 | 5.91 | 4.19 | 4.24 | 3.52 |
| 19:05 | 915 | 829 | 767 | 691 | 5.44 | 4.47 | 3.85 | 3.3 |
| 19:10 | 834 | 774 | 687 | 660 | 4.93 | 4.23 | 3.36 | 3.29 |
| 19:15 | 822 | 722 | 655 | 604 | 5.14 | 4.04 | 3.37 | 3.06 |
| 19:20 | 742 | 674 | 594 | 592 | 4.68 | 3.9 | 3.1 | 3.27 |
| 19:25 | 734 | 618 | 541 | 466 | 4.91 | 3.65 | 2.87 | 2.32 |
| 19:30 | 705 | 564 | 551 | 504 | 4.87 | 3.36 | 3.23 | 2.96 |
| 19:35 | 637 | 555 | 541 | 478 | 4.38 | 3.46 | 3.33 | 2.9 |
| 19:40 | 651 | 518 | 454 | 390 | 4.66 | 3.23 | 2.59 | 2.15 |
| 19:45 | 626 | 537 | 479 | 411 | 4.48 | 3.5 | 2.91 | 2.43 |
| 19:50 | 625 | 506 | 446 | 401 | 4.51 | 3.21 | 2.61 | 2.37 |
| 19:55 | 642 | 488 | 444 | 376 | 4.67 | 3.03 | 2.6 | 2.11 |
| 20:00 | 597 | 495 | 440 | 386 | 4.21 | 3.09 | 2.54 | 2.2 |

**Table 2. Average delay and average pedestrian crossing wait times.**

| Time | Average Delay (s) | | | | Average pedestrian crossing wait times(s) | | | |
| --- | --- | --- | --- | --- | --- | --- | --- | --- |
|  | Webster | DQN | DDQN | EXP-DDQN | Webster | DQN | DDQN | EXP-DDQN |
| 06:00 | 9.79 | 6.99 | 7.12 | 5.4 | 10.69 | 13.74 | 8.96 | 5.72 |
| 06:05 | 9.67 | 7.27 | 6.73 | 6.52 | 14.81 | 12.27 | 8.39 | 4.84 |
| 06:10 | 10.72 | 7.75 | 6.94 | 6.23 | 13.55 | 12.07 | 7.75 | 4 |
| 06:15 | 11.85 | 8.77 | 8.23 | 7.55 | 15.27 | 13.44 | 8.16 | 8.71 |
| 06:20 | 10.9 | 9.56 | 8.69 | 7.75 | 15.04 | 13.7 | 8.21 | 9.81 |
| 06:25 | 11.37 | 9.98 | 8.55 | 8.06 | 14.92 | 12.1 | 10.74 | 8.43 |
| 06:30 | 13.28 | 10.88 | 9.04 | 8 | 19.39 | 14.11 | 10.27 | 4.72 |
| 06:35 | 13.09 | 10.69 | 10.07 | 8.72 | 18.88 | 15.52 | 9.76 | 9 |
| 06:40 | 12.55 | 12.29 | 9.16 | 10.23 | 15.74 | 16.64 | 11.49 | 10.93 |
| 06:45 | 13.77 | 11.33 | 9.61 | 10.44 | 18.79 | 16.39 | 11.41 | 9.89 |
| 06:50 | 13.35 | 14.1 | 10.54 | 9.64 | 19.47 | 17.04 | 11.37 | 13.24 |
| 06:55 | 13.68 | 12.75 | 11.28 | 10.83 | 20.2 | 16.21 | 18.42 | 11.92 |
| 07:00 | 14.53 | 11.85 | 11.98 | 11.64 | 21.96 | 17.51 | 16.33 | 12.78 |
| 07:05 | 13.02 | 11.9 | 13.13 | 9.92 | 22.05 | 21.78 | 16.82 | 13.85 |
| 07:10 | 13.33 | 13.29 | 12.79 | 11.84 | 22.23 | 20 | 18.64 | 17.03 |
| 07:15 | 14.34 | 12.81 | 12.06 | 10.96 | 23.53 | 18.77 | 18.21 | 15.6 |
| 07:20 | 13.98 | 13.57 | 12.18 | 11.95 | 22.43 | 20.07 | 18.26 | 16.74 |
| 07:25 | 14.98 | 13.31 | 11.33 | 10.82 | 24.53 | 25.04 | 20.29 | 16.59 |
| 07:30 | 13.86 | 12.73 | 11.97 | 9.85 | 26.87 | 26.27 | 19.91 | 19.11 |
| 07:35 | 13.25 | 11.91 | 11.55 | 9.78 | 26.63 | 25.13 | 21.43 | 17.94 |
| 07:40 | 15.29 | 11.1 | 11.77 | 10.85 | 27.91 | 26.8 | 23.7 | 21.18 |
| 07:45 | 13.61 | 11.63 | 10.53 | 10.7 | 28.18 | 25.48 | 24.28 | 21.46 |
| 07:50 | 13.47 | 12.3 | 11.23 | 9.39 | 27.39 | 24.1 | 25.03 | 21.01 |
| 07:55 | 11.87 | 11.38 | 11.63 | 10.22 | 28.42 | 26.3 | 20.2 | 20.81 |
| 08:00 | 12.14 | 9.77 | 9.88 | 7.94 | 28.13 | 28.42 | 23.31 | 18.83 |
| 08:05 | 12.2 | 10.45 | 9.84 | 8.76 | 24.46 | 27.65 | 24.15 | 20.74 |
| 08:10 | 10.73 | 10.16 | 9.6 | 7.38 | 25.22 | 27.6 | 25.67 | 20.03 |
| 08:15 | 11.48 | 8.68 | 8.26 | 7.36 | 26.96 | 25.05 | 20.46 | 21.79 |
| 08:20 | 10.25 | 9.05 | 8.31 | 7.46 | 26.58 | 25.42 | 21.26 | 19.93 |
| 08:25 | 10.05 | 8.53 | 7.69 | 6.3 | 25.04 | 23.93 | 20.88 | 19.95 |
| 08:30 | 9.38 | 7.15 | 7.34 | 6.26 | 26.55 | 22.33 | 21.13 | 18.53 |
| 08:35 | 10.95 | 7.95 | 6.25 | 6.97 | 25.28 | 26.71 | 18.26 | 17.16 |
| 08:40 | 9.09 | 7.75 | 6.52 | 5.34 | 24.99 | 23.08 | 18.87 | 17.29 |
| 08:45 | 7.92 | 7.84 | 6.57 | 6.14 | 23.68 | 20.13 | 17.09 | 15.09 |
| 08:50 | 9.13 | 7.51 | 7.03 | 4.27 | 21.78 | 21.1 | 16.87 | 15.22 |
| 08:55 | 7.22 | 5.3 | 6.37 | 5.33 | 18.57 | 19.46 | 18.56 | 13.83 |
| 09:00 | 8.13 | 5.42 | 7.09 | 5.82 | 20.09 | 18.34 | 14.73 | 11.74 |
| 09:05 | 6.19 | 6.37 | 4.55 | 2.48 | 19.92 | 17.59 | 15.87 | 11.83 |
| 09:10 | 6.52 | 6.2 | 5.68 | 3.65 | 19.36 | 15.08 | 16.54 | 11.5 |
| 09:15 | 7.59 | 6.05 | 4.98 | 4.6 | 18.77 | 17.61 | 14.59 | 12.04 |
| 09:20 | 7.9 | 8.59 | 6.46 | 3.85 | 18.5 | 15.56 | 11.12 | 10 |
| 09:25 | 7.35 | 5.87 | 3.96 | 4.21 | 15.71 | 16.55 | 11.41 | 7.68 |
| 09:30 | 7.03 | 6.23 | 3.85 | 3.34 | 16.02 | 12.74 | 10.55 | 10.01 |
| 09:35 | 6.82 | 6.02 | 3.98 | 3.83 | 15.75 | 12.88 | 11.69 | 7.42 |
| 09:40 | 5.82 | 5.73 | 2.71 | 3.58 | 15.82 | 11.51 | 8.55 | 6.77 |
| 09:45 | 6.39 | 4.91 | 3.94 | 4.6 | 13.52 | 13.83 | 11.61 | 4.14 |
| 09:50 | 6.57 | 5.74 | 3.73 | 3.84 | 14.62 | 11.26 | 9.24 | 4.63 |
| 09:55 | 7.76 | 4.5 | 4.43 | 3.88 | 14.09 | 12.27 | 8.84 | 10.5 |
| 10:00 | 7.18 | 4.91 | 4.57 | 3.27 | 15.48 | 14.23 | 10.04 | 6.01 |
| 10:05 | 5.49 | 4.71 | 5.8 | 3.21 | 14.98 | 11.2 | 9.25 | 7.63 |
| 10:10 | 7.16 | 5.16 | 5.06 | 3.25 | 14.82 | 12.33 | 11.14 | 6.26 |
| 10:15 | 6.6 | 6.96 | 3.84 | 3.92 | 13.45 | 9.78 | 12.09 | 8.98 |
| 10:20 | 6.38 | 3.62 | 3.6 | 3.28 | 18.16 | 10.84 | 9.53 | 7.26 |
| 10:25 | 7.43 | 5.69 | 4.73 | 3.87 | 16.18 | 13.28 | 12 | 7.3 |
| 10:30 | 7.79 | 3.87 | 3.31 | 5.32 | 15.17 | 15.89 | 9.16 | 7.43 |
| 10:35 | 7.74 | 4.82 | 5.86 | 4.39 | 17.21 | 13.33 | 11.44 | 8.6 |
| 10:40 | 6.36 | 6.11 | 5.38 | 3.47 | 18.67 | 15.13 | 12.66 | 10.48 |
| 10:45 | 6.83 | 5.33 | 4.11 | 4.74 | 18.86 | 16.66 | 9.35 | 9.31 |
| 10:50 | 7.4 | 4.47 | 3.17 | 3.51 | 24 | 13.91 | 17.54 | 11.17 |
| 10:55 | 7.98 | 4.83 | 5.68 | 2.27 | 18.91 | 16.5 | 14.07 | 11.06 |
| 11:00 | 6.89 | 6.02 | 4.58 | 3.17 | 21.88 | 15.24 | 14.75 | 15.1 |
| 11:05 | 7.21 | 4.97 | 5.75 | 2.56 | 22.41 | 18.35 | 12.14 | 12.27 |
| 11:10 | 6.57 | 5.83 | 3.58 | 3.87 | 20.89 | 16.73 | 14.67 | 16.03 |
| 11:15 | 6.6 | 5.79 | 4.48 | 4.27 | 21.3 | 20.46 | 16.39 | 15.11 |
| 11:20 | 8.32 | 5.35 | 5.08 | 5.71 | 22.33 | 20.52 | 15.72 | 15.03 |
| 11:25 | 8.88 | 7.71 | 5.24 | 4.76 | 22.49 | 19.57 | 16.9 | 14.02 |
| 11:30 | 7.88 | 6.64 | 4.98 | 4.46 | 19.86 | 17 | 16.42 | 16.48 |
| 11:35 | 8.89 | 4.66 | 5.98 | 5.45 | 23.55 | 18.39 | 19.44 | 15.99 |
| 11:40 | 8.53 | 6.59 | 4.78 | 3.17 | 25.92 | 20.53 | 19.84 | 15.75 |
| 11:45 | 7.89 | 6.07 | 5.69 | 5.29 | 24.85 | 21.14 | 17.7 | 15.59 |
| 11:50 | 8.86 | 7.45 | 6.07 | 5.89 | 24.44 | 23.65 | 18.94 | 13.21 |
| 11:55 | 9.98 | 6.31 | 6.56 | 4.26 | 27.97 | 21.63 | 17.96 | 17.33 |
| 12:00 | 8.89 | 7.03 | 6.89 | 6.54 | 25.67 | 23.33 | 17.8 | 15.07 |
| 12:05 | 10.35 | 7.7 | 5.6 | 6.07 | 25.12 | 23.65 | 20.65 | 14.67 |
| 12:10 | 7.18 | 8.17 | 5.45 | 5.64 | 24.73 | 21.59 | 17.16 | 18.08 |
| 12:15 | 10.1 | 6.68 | 7.86 | 6.65 | 25.52 | 24.76 | 17.08 | 16.36 |
| 12:20 | 9.67 | 7.53 | 7.27 | 8.12 | 23.54 | 20.95 | 17.55 | 16.11 |
| 12:25 | 9.51 | 7.57 | 6.55 | 6.6 | 25.01 | 18.94 | 16.07 | 15.94 |
| 12:30 | 9.96 | 7.57 | 8.53 | 6.79 | 21.74 | 22 | 17.61 | 14.37 |
| 12:35 | 8.42 | 9.62 | 7.5 | 6.34 | 23.31 | 16.53 | 15.41 | 13.21 |
| 12:40 | 9.94 | 8.63 | 8.45 | 6.13 | 23.3 | 18.07 | 16.3 | 13.09 |
| 12:45 | 10.48 | 7.38 | 7.65 | 7.56 | 21.5 | 21.14 | 15.15 | 13.08 |
| 12:50 | 11.43 | 9.19 | 9.3 | 6.27 | 19.75 | 18.37 | 14.06 | 14.58 |
| 12:55 | 9.87 | 10.19 | 9.09 | 7.05 | 22.22 | 17.16 | 14.23 | 14.29 |
| 13:00 | 9.65 | 9.33 | 7.5 | 6.62 | 21.39 | 16.05 | 13.78 | 10.99 |
| 13:05 | 9.89 | 7.27 | 8.47 | 7.37 | 21.56 | 15.61 | 14.4 | 12.68 |
| 13:10 | 10.98 | 8.06 | 8.17 | 7.22 | 20.38 | 15.93 | 14.47 | 12.32 |
| 13:15 | 10.46 | 9.41 | 8.69 | 7.72 | 16.54 | 13.92 | 13.7 | 8.09 |
| 13:20 | 9.69 | 7.74 | 6.74 | 6.4 | 18.15 | 15.04 | 12.37 | 9.79 |
| 13:25 | 10.42 | 8.56 | 7.96 | 6.49 | 17.66 | 15.54 | 12.85 | 10.41 |
| 13:30 | 9.97 | 8.71 | 8.14 | 5.81 | 15.59 | 14.21 | 9.25 | 9.6 |
| 13:35 | 10.53 | 7.21 | 5.75 | 6.1 | 14.77 | 15.26 | 11.35 | 6.71 |
| 13:40 | 9.04 | 7.76 | 6.06 | 6.6 | 16.32 | 13.26 | 11.97 | 8.1 |
| 13:45 | 9.18 | 5.05 | 5.21 | 6.75 | 14.02 | 11.67 | 12.91 | 6.42 |
| 13:50 | 8.96 | 6.65 | 6.46 | 5.24 | 12.73 | 13.38 | 10.32 | 5.46 |
| 13:55 | 7.93 | 7.1 | 7.07 | 6.5 | 13.58 | 14.21 | 10.52 | 4.18 |
| 14:00 | 9.16 | 6.13 | 7.53 | 6.71 | 10.1 | 11.94 | 9.21 | 9.34 |
| 14:05 | 8.96 | 8.25 | 6.21 | 5.78 | 14.82 | 12.06 | 8.14 | 8.83 |
| 14:10 | 8.58 | 5.63 | 7.28 | 6.78 | 13.59 | 10.94 | 6.85 | 6.02 |
| 14:15 | 8.22 | 6.25 | 4.7 | 4.49 | 12.22 | 13.78 | 7.53 | 6.05 |
| 14:20 | 7.11 | 6.55 | 4.28 | 3.95 | 11.73 | 12.79 | 8.74 | 8.22 |
| 14:25 | 7.75 | 7.44 | 5.45 | 3.37 | 14.51 | 12.17 | 9.16 | 6.82 |
| 14:30 | 7.67 | 5 | 5.65 | 5.84 | 15.68 | 9.85 | 8.19 | 8 |
| 14:35 | 7.17 | 6.95 | 5.19 | 5.04 | 12.83 | 10.45 | 8.44 | 3.12 |
| 14:40 | 7.56 | 5.9 | 3.44 | 4.35 | 12.8 | 11.63 | 10.06 | 8.21 |
| 14:45 | 7.91 | 5 | 4.91 | 4.51 | 11.47 | 10.19 | 9.56 | 6.3 |
| 14:50 | 9 | 6.06 | 3.85 | 3.29 | 11.82 | 11.17 | 9.13 | 5.99 |
| 14:55 | 7.55 | 5.77 | 5.34 | 6.07 | 15.77 | 10.64 | 9.62 | 7.34 |
| 15:00 | 7.55 | 5.06 | 5.03 | 4.14 | 14.65 | 11.93 | 10.28 | 8.37 |
| 15:05 | 7.23 | 5.54 | 3.94 | 4.08 | 14.39 | 12.78 | 9.22 | 7.97 |
| 15:10 | 5.72 | 5.14 | 4.24 | 4.53 | 13.29 | 12.82 | 10.3 | 8.24 |
| 15:15 | 7.21 | 5.52 | 3.78 | 4.31 | 15.97 | 9.23 | 11.67 | 10.37 |
| 15:20 | 7.27 | 5.95 | 4.57 | 4.1 | 14.99 | 13.43 | 7.76 | 6.64 |
| 15:25 | 9.2 | 6.7 | 5.4 | 3.3 | 17.11 | 14.29 | 12.51 | 4.59 |
| 15:30 | 7.11 | 4.47 | 3.88 | 4.34 | 13.47 | 15.39 | 12.04 | 8.3 |
| 15:35 | 7.56 | 7.22 | 5.12 | 5.52 | 17.39 | 13.9 | 11.39 | 9.82 |
| 15:40 | 7.36 | 4.02 | 4.36 | 5.16 | 17.08 | 15.79 | 12.18 | 9.93 |
| 15:45 | 6.55 | 5.56 | 4.25 | 5.45 | 18.07 | 16.58 | 11.7 | 10.59 |
| 15:50 | 8.51 | 6.27 | 4.91 | 3.89 | 17.47 | 17.13 | 12.07 | 10.05 |
| 15:55 | 8.35 | 6.17 | 4.32 | 3.66 | 20.72 | 17.73 | 14.58 | 12.05 |
| 16:00 | 8.56 | 5.62 | 4.88 | 4.52 | 19.07 | 19.95 | 12.75 | 12.33 |
| 16:05 | 7.4 | 6.16 | 4.57 | 4.87 | 21.95 | 19.59 | 16.16 | 10.06 |
| 16:10 | 9.49 | 6.17 | 7.34 | 5.94 | 20.75 | 21.9 | 15.95 | 14.41 |
| 16:15 | 7.52 | 6.37 | 6.07 | 3.99 | 23.79 | 20.63 | 12.95 | 13.98 |
| 16:20 | 9.42 | 7.83 | 5.79 | 6.87 | 21.61 | 20.62 | 16.68 | 13.08 |
| 16:25 | 11.05 | 7.78 | 6.87 | 5.87 | 21.73 | 22.31 | 16.59 | 15.89 |
| 16:30 | 8.88 | 7.32 | 6.98 | 6.03 | 24.18 | 21.39 | 17.02 | 14.02 |
| 16:35 | 9.63 | 9 | 7.31 | 5.98 | 23.64 | 24.84 | 18.15 | 17.42 |
| 16:40 | 10.61 | 8.97 | 8.42 | 5.9 | 25.48 | 21.7 | 20.45 | 16.81 |
| 16:45 | 10.6 | 9.85 | 9.01 | 8.36 | 27.67 | 25.44 | 19.58 | 21.57 |
| 16:50 | 10.26 | 10.2 | 8.47 | 8.26 | 27.34 | 25 | 21.51 | 20.24 |
| 16:55 | 12.07 | 9.55 | 8.95 | 7.68 | 27.06 | 27.55 | 22.2 | 18.17 |
| 17:00 | 11.69 | 10.29 | 9.72 | 8.2 | 27.48 | 23.08 | 20.91 | 16.62 |
| 17:05 | 13.45 | 11.87 | 8.63 | 9.5 | 27.85 | 27.83 | 22.82 | 22.66 |
| 17:10 | 12.86 | 12.29 | 9.79 | 11.63 | 30.09 | 26.72 | 24.97 | 23.9 |
| 17:15 | 15.36 | 12.3 | 12.61 | 10.61 | 30.48 | 25.81 | 24.83 | 19.09 |
| 17:20 | 13.99 | 12.91 | 13.33 | 10.11 | 29.59 | 28.57 | 25.2 | 25.49 |
| 17:25 | 14.82 | 14.3 | 12.28 | 11.58 | 31.5 | 29.18 | 24.31 | 25.63 |
| 17:30 | 16.15 | 13.23 | 13.36 | 11.99 | 27.9 | 29.83 | 21.59 | 25.7 |
| 17:35 | 14.9 | 14.52 | 13.53 | 10.42 | 30.9 | 29.29 | 25.74 | 24.26 |
| 17:40 | 16.38 | 14.24 | 16.06 | 12.86 | 31.97 | 29.42 | 26.96 | 23.42 |
| 17:45 | 17.5 | 14.48 | 14.75 | 12.97 | 33.07 | 28.32 | 28.33 | 26.11 |
| 17:50 | 15.36 | 15.73 | 13.94 | 13.9 | 31.14 | 27.39 | 26.08 | 27.58 |
| 17:55 | 16.91 | 15.62 | 13.4 | 14.94 | 31.71 | 30.49 | 27.97 | 24.84 |
| 18:00 | 17.01 | 15.65 | 12.91 | 14.4 | 33.25 | 29.35 | 27.56 | 25.07 |
| 18:05 | 17.39 | 16.01 | 14.32 | 13.25 | 31.7 | 31.54 | 25.74 | 22.77 |
| 18:10 | 15.66 | 14.86 | 13.44 | 12.46 | 31.08 | 30.88 | 27.23 | 25.63 |
| 18:15 | 15.4 | 15.2 | 12.72 | 15.22 | 33.7 | 31.48 | 26.93 | 23.68 |
| 18:20 | 16.62 | 14.15 | 13.08 | 12.95 | 31.97 | 31.27 | 25.88 | 25.57 |
| 18:25 | 16.12 | 14.34 | 12.42 | 12.59 | 33.28 | 29.31 | 25.55 | 26.82 |
| 18:30 | 15.7 | 13.6 | 14.25 | 12.18 | 26.29 | 27.15 | 24.94 | 23.36 |
| 18:35 | 15.35 | 13.36 | 13.18 | 11.94 | 32.06 | 26.79 | 25.48 | 20.89 |
| 18:40 | 14.07 | 13.29 | 12.01 | 11.2 | 29.84 | 27.9 | 25.37 | 23.16 |
| 18:45 | 14.3 | 11.66 | 12.7 | 10.36 | 27.41 | 27.72 | 23.64 | 24.36 |
| 18:50 | 13.83 | 13.47 | 11.06 | 9.86 | 30.34 | 25.95 | 24.5 | 20.25 |
| 18:55 | 12.5 | 10.47 | 9.78 | 9.74 | 29.16 | 25.84 | 21.05 | 19.9 |
| 19:00 | 14.03 | 9.77 | 11.16 | 8.8 | 25.03 | 24.74 | 22.62 | 18.27 |
| 19:05 | 12.39 | 11.14 | 9.84 | 8.14 | 27.48 | 24.24 | 21.6 | 19.9 |
| 19:10 | 10.54 | 10.33 | 8.06 | 8.28 | 28.26 | 24.1 | 24.04 | 22.11 |
| 19:15 | 11.52 | 9.7 | 8.25 | 7.49 | 24.88 | 23.53 | 21.65 | 17.28 |
| 19:20 | 9.74 | 9.23 | 7.22 | 8.43 | 25.67 | 19.94 | 21.7 | 16.62 |
| 19:25 | 10.71 | 8.27 | 6.37 | 4.66 | 26.11 | 21 | 18.87 | 15.69 |
| 19:30 | 10.59 | 7.15 | 7.81 | 7.24 | 24.69 | 20.02 | 18.24 | 14.62 |
| 19:35 | 8.63 | 7.54 | 8.21 | 6.98 | 22.66 | 21.59 | 18.4 | 16.1 |
| 19:40 | 9.71 | 6.6 | 5.22 | 3.98 | 23.29 | 19.72 | 17.3 | 13.12 |
| 19:45 | 8.95 | 7.6 | 6.47 | 5.05 | 23.45 | 20.34 | 17.76 | 17.5 |
| 19:50 | 9 | 6.43 | 5.22 | 4.75 | 21.94 | 19.02 | 15.69 | 11.63 |
| 19:55 | 9.62 | 5.64 | 5.11 | 3.67 | 19.28 | 17.91 | 15.74 | 15.03 |
| 20:00 | 7.69 | 5.83 | 4.81 | 3.96 | 18.34 | 17.26 | 12.36 | 11.44 |
